# Mathematically mapping the network of cells in the tumor microenvironment

**DOI:** 10.1016/j.crmeth.2025.100985

**Published:** 2025-02-14

**Authors:** Mike van Santvoort, Óscar Lapuente-Santana, Maria Zopoglou, Constantin Zackl, Francesca Finotello, Pim van der Hoorn, Federica Eduati

**Affiliations:** 1 Department of Mathematics and Computer Science, Eindhoven University of Technology, PO Box 513, Eindhoven 5600MB, the Netherlands; 2 Institute for Complex Molecular Systems, Eindhoven University of Technology, PO Box 513, Eindhoven 5600MB, the Netherlands; 3 Department of Biomedical Engineering, Eindhoven University of Technology, PO Box 513, Eindhoven 5600MB, the Netherlands; 4 Bioinformatics Unit, Spanish National Cancer Research Centre (CNIO), 28029 Madrid, Spain; 5 Department of Molecular Biology, Digital Science Center (DiSC), University of Innsbruck, 6020 Innsbruck, Austria

**Keywords:** cell-cell networks, random graphs, bulk transcriptomics, ligand-receptor interactions, patient-specific models, tumor microenvironment, immunotherapy

## Abstract

Cell-cell interaction (CCI) networks are key to understanding disease progression and treatment response. However, existing methods for inferring these networks often aggregate data across patients or focus on cell-type level interactions, providing a generalized overview but overlooking patient heterogeneity and local network structures. To address this, we introduce “random cell-cell interaction generator” (RaCInG), a model based on random graphs to derive personalized networks leveraging prior knowledge on ligand-receptor interactions and bulk RNA sequencing data. We applied RaCInG to 8,683 cancer patients to extract 643 network features related to the tumor microenvironment and unveiled associations with immune response and subtypes, enabling prediction and explanation of immunotherapy responses. RaCInG demonstrated robustness and showed consistencies with state-of-the-art methods. Our findings highlight RaCInG’s potential to elucidate patient-specific network dynamics, offering insights into cancer biology and treatment responses. RaCInG is poised to advance our understanding of complex CCI s in cancer and other biomedical domains.

## Highlights

• RaCInG infers patient-specific cell-cell networks from bulk transcriptomics
• RaCInG provides a user-friendly, modular pipeline for network inference
• RaCInG is robust and mathematically tractable
• Extracted features reveal insights into tumors and immune response

## Motivation

While numerous methods exist for inferring CCI networks, most rely on single-cell transcriptomics, which are not yet widely accessible. In contrast, bulk transcriptomics data, increasingly available in clinical settings, remain underutilized for this purpose. To bridge this gap, we developed random cell-cell interaction generator, a tool that leverages current general knowledge on cellular interactions and integrates it with patient transcriptomics data to derive patient-specific cell-cell networks. The extracted network features go beyond direct cell-cell communication, capturing broader intercellular dynamics that provide deeper insights into patient-specific disease mechanisms and therapeutic responses.

## Graphical abstract

**Figure:**
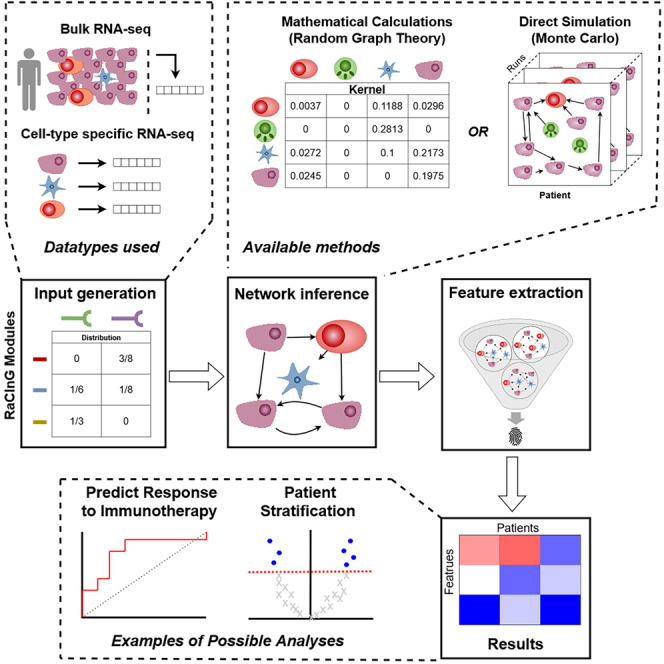

## Teaser

Van Santvoort et al. develop a mathematical model to infer CCI networks from bulk transcriptomics data, which is widely available for cancer patients. They show that, despite the lower resolution of bulk data, the model uncovers how cells in a tumor communicate and predicts potential treatment efficacy.

## Introduction

Stratifying patients based on tumor characteristics is crucial for predicting treatment responses. Understanding the tumor microenvironment (TME), composed of cells and molecules, is essential for this process. While major breakthroughs have focused on individual components, such as programmed cell death ligand 1 blockers^1^ to counteract the molecule’s pro-tumor effect,^2^ the TME’s behavior cannot be explained by studying components in isolation.^3^^,^^4^ For instance, proteins like tumor necrosis factor-α exhibit pro- or anti-tumor effects depending on the context,^5^ necessitating a holistic approach to the TME.

Modeling the TME as a cell-cell interaction (CCI) network, often inferred from RNA sequencing (RNA-seq) data,^6^ offers an unbiased perspective. Such networks have been used to understand cell crosstalk in the TME^7^^,^^8^^,^^9^^,^^10^^,^^11^^,^^12^; however, they are often limited by their low-resolution focus on cell- and protein-type levels,^6^^,^^7^^,^^8^^,^^9^ masking local network structures and failing to capture patient-specific communication patterns.^11^^,^^13^ High-resolution methods using single-cell RNA-seq data^9^^,^^12^ overcome this but face challenges, including technical limitations (greater uncertainty, drop-outs, and distortion of true cellular proportions), high costs, and difficulties in sample preparation limiting clinical applicability.^14^ Recent probabilistic approaches using bulk RNA-seq data reconstruct CCI networks for individual patients,^10^ but operate on a cell type level, providing only a mean-field approximation that lacks the ability to explode detailed TME network features.

Random graphs models^15^ can address these limitations, leveraging stochasticity to fill knowledge gaps without introducing bias. Despite explicitly adding noise in network construction, emergent network behavior often remains statistically consistent and could serve as fingerprints of the underlying network. This approach enables the inference of local properties from high-resolution networks, even when direct reconstruction is unfeasible.

Here, we present “random cell-cell interaction generator” (RaCInG), a modular toolbox, that extends the application of random graph models to reconstruct single-cell interaction networks by integrating widely accessible bulk RNA-seq data with prior knowledge. By adhering to biological constraints and resolving uncertainties through uniform random sampling,^16^ our method avoids additional assumptions while uncovering critical network features of the TME. We validate RaCInG analyzing the TME networks for more than 8,000 cancer patients, demonstrating robust properties predictive of immune response scores, immune subtype and of response to immune checkpoint blockers (ICBs).

## Results

### RaCInG: A modular pipeline for CCI network inference

**Figure 1:**
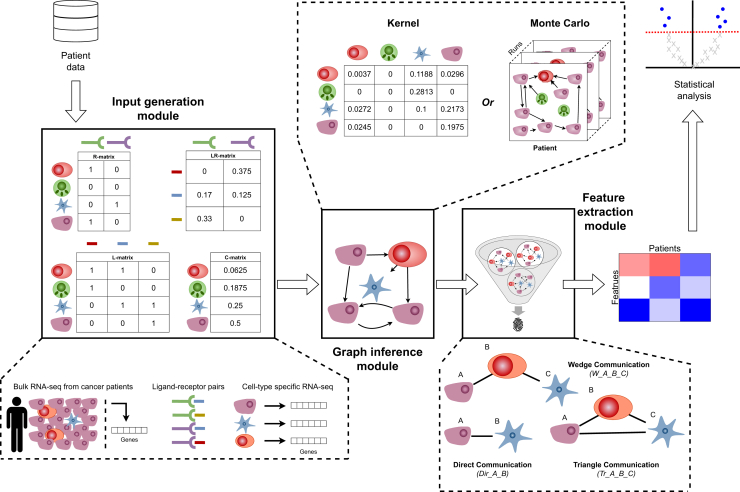
Overview of the RaCInG modular pipeline The “input generation module” uses bulk RNA-seq data to deconvolve cell fractions (C-matrix) and quantify ligand-receptor (LR) pairs (LR-matrix), based on prior knowledge of possible LR interactions. It also uses cell type-specific gene expression to determine ligands and receptors possibly expressed by each cell type (L- and R-matrices). These (or alternative user-defined) four input matrices are then used by RaCInG to infer CCIs through either kernel calculations or Monte Carlo simulations in the “graph inference module.” In the “feature extraction module,” pre-defined features from each inferred network serve as patient-specific CCI fingerprints, including local (direct, wedge, and triangle communication), and global (GSCC and degree centrality) features computed considering individual cells of a given type. See also Figure S1.

RaCInG is a modular pipeline to infer personalized CCI networks on an individual cell level from patients’ bulk RNA-seq data. RaCInG consists of three modules, which are illustrated in Figure 1. Briefly, the pipeline consists of the input generation module, to prepare the RaCInG inputs by integrating the bulk RNA-seq data with prior knowledge on ligands and receptors; the graph inference module to generate patient-specific networks; and the feature extraction module to extract relevant network properties that can be used as patient-specific fingerprints for downstream statistical analysis.

The graph inference module forms the core of RaCInG. The input generation and the feature extraction modules are provided as tools to prepare and analyze the data, respectively, but they can be adapted by the users based on their needs. Some examples of alternative ways to prepare the input data are also provided in this paper.

### Input generation module

To generate personalized networks, RaCInG requires information on (1) the cellular composition of the sample, encoded in the cell-type matrix (C-matrix); (2) The probability of ligand-receptor binding in that sample, encoded in the ligand-receptor matrix (LR-matrix); and (3 and 4) information about which ligands and receptors can be expressed by each cell type, encoded in the ligand- and receptor-compatibility matrices (L- and R-matrix).

The input generation module can derive these four matrices (Figure 1) requiring only the patients’ bulk RNA-seq data (details in the STAR methods). The C-matrix is derived by applying an ensemble of deconvolution algorithms to the bulk RNA-seq samples to estimate cell fractions of a user-specified set of cell types using the immunedeconv package.^17^ Different deconvolution methods to derive cell fractions could also be used; we provide in this paper an analysis of the impact of variations in the C-matrix on the generated graphs. The LR-matrix is defined based on a list of 971 literature-curated LR interactions derived from OmniPath^18^ and quantified for each patient as the most limiting factor between the expression of the ligand and the receptor based on the RNA-seq data.^19^ The L- and R-matrices were defined as general (i.e., not patient specific) knowledge on which ligands and which receptors can be expressed by a specific cell type based on cell type-specific gene expression data. An alternative approach used in this work is to derive patient-specific L- and R-matrices applying expression purification algorithms to bulk RNA-seq data.

### Graph inference module

Using the previously described four input matrices, RaCInG provides two ways to derive patient-specific networks: generating networks directly using Monte Carlo simulations or summarizing the underlying random graph model using a kernel-based method. Two additional parameters are required as input: the number of cells in the CCI network (N; only for Monte Carlo method) and the average number of ligand-receptor interactions per cell (λ; both methods).

In the Monte Carlo method, we generate networks by (1) assigning cell types to N nodes randomly based on C-matrix probabilities, (2) assigning types to λN LR interactions randomly based on the LR-matrix probabilities, and (3) assigning, for each LR interaction, the ligand and the receptor to a random compatible cell, based on the L- and R-matrices, and connecting the corresponding nodes with an arc (more details in STAR methods and Figure S1A). Since Monte Carlo simulations are stochastic, the process is repeated multiple times to generate an ensemble of networks for each patient.

In the kernel method, we directly leverage random graph theory^20^ to calculate a patient-specific function (called the kernel of RaCInG; see STAR methods) that encodes the probability of an interaction emerging between two cell-types.

Both Monte Carlo and kernel methods generate CCI representations compatible with RNA-seq data and prior knowledge without introducing bias. Monte Carlo is computationally heavier but constructs explicit networks for the extraction of a multitude of robust features. The kernel method is faster, but requires mathematical proofs to validate the extracted features.^20^^,^^21^

In our case studies, we primarily apply the kernel method for efficiency, relying on the Monte Carlo method only when equivalence proofs are unavailable.^15^^,^^20^^,^^21^

### Feature extraction module

After graph inference, features are extracted either from the ensemble of graphs (Monte Carlo method), or directly from the kernels (kernel method) (STAR methods).

Our analysis of CCIs in the TME focused on five feature types. Three were (undirected) graph motifs: the number of direct interactions (named *Dir_A_B*, for a given pair of cell types A and B), the number of wedges (*W_A_B_C*, given cell types A, B and C) and the number of triangles (*Tr_A_B_C*) between cells with a given type (Figure 1, STAR methods). Direct interactions measure how often individual cells of given types directly communicate through ligand-receptor interactions. Wedges and triangles quantify communications of individual cells in specific network motifs, allowing to contextually investigate cellular communication (e.g., which cluster of three cells most often communicate together in case of triangles). The remaining two feature types were global graph features: the fraction of each cell type in the giant strongly connected component (GSCC) and their degree centrality (STAR methods). The GSCC represents the largest cluster of cells with mutual communication paths, indicating which cell types form the core of TME communication. Degree centrality measures the connectivity of each cell type in the network, capturing the extent to which a cell type interacts with others (out-degree centrality) or is interacted upon by others (in-degree centrality). It serves as an indicator of its central role in the TME. RaCInG users can define more/different features and easily extend the model to incorporate them.

To address bias from cell type abundances, RaCInG normalizes features. Since LR interaction assignment is random, highly abundant cell types are more likely to interact, influencing feature values. To account for this, RaCInG recomputes the network features for each patient using a uniform LR-matrix, where all LR pairs have equal probability. The normalized features are computed as the fold-change between the feature values obtained using the data-derived versus uniform LR-matrix (Figure S1D, STAR methods). This ensures that all feature values are comparable between samples with different cellular composition, as all values are scaled to the same magnitude (compare Tables S1 and S2). Unless specified otherwise, all kernel values and features are reported as normalized values.

### Application to studying the TME

As a case study we used RaCInG to study CCIs in the TME of 8,562 cancer patients from The Cancer Genome Atlas (TCGA) and 121 patients treated with ICB therapy (see STAR methods for more details on the patient cohorts). We focused on nine different cell types (Table 1), resulting in a total of 643 features. These features include 45 direct communications, 405 wedges, 165 triangles, 10 GSCCs (one of each cell type and one aggregating all cell types), and 18 degree centralities (9 in-degree and 9 out-degree centralities). Note that the number of features depends on the user defined cell types.

**Table 1:** Cell types included in case studies

Cell name	Abbreviation	Cell name	Abbreviation
Tumor	Tumor	B cell	B
Cancer associated fibroblast	CAF	macrophage	M
Endothelial cell	Endo	dendritic cell	DC
CD8^+^ T cell	CD8	regulatory T cell	Treg
Natural killer cell	NK	–	–

In the next sections, we used the case study first to investigate the robustness of RaCInG to uncertainties in the input matrices. We assessed the impact of the deconvolution (C-matrix) and of the prior knowledge of expressed ligands and receptors (L- and R-matrices) on the resulting networks. We did not directly assess the effect of the LR-matrix, as it has only a linear effect on the kernel; therefore, we may directly expect RaCInG to be stable with respect to deviations in this matrix (STAR methods). Then, we validated the resulting networks by comparing them with state-of-the-art techniques. Finally, we used the networks to study how cell-cell communication is associated with immune subtypes, immune response, and response to immunotherapy.

### RaCInG is robust to cellular decomposition

**Figure 2:**
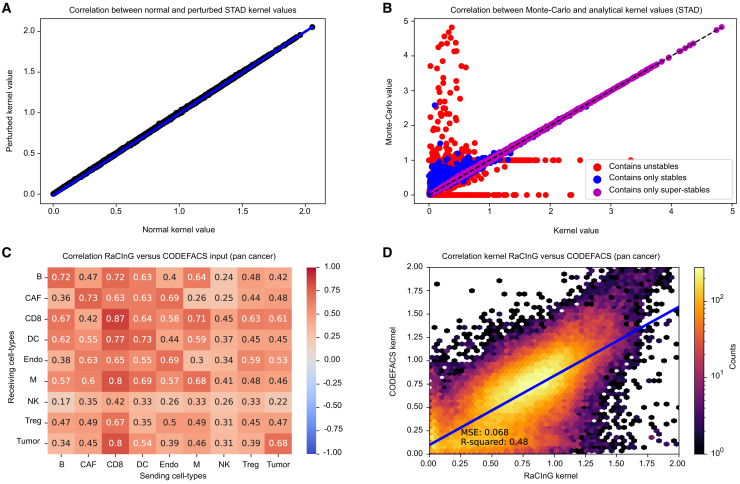
Robustness of RaCInG (A) Sensitivity of RaCInG kernel for perturbations of the C-matrix, with tumor cell quantification directly perturbed, and other cell-types renormalized (maximally 10%; STAD TCGA dataset; 33,615 kernel values). (B) Agreement between the kernel and Monte Carlo methods for unstable, stable and super-stable features (STAD TCGA dataset; 16,8075 wedges). (C) Pearson r correlation between kernel values from general L- and R-matrices from the RaCInG pipeline vs. the patient-specific ones obtained using CODEFACS (STAD + SKCM TCGA analysis; 71,442 kernel values). (D) Linear regression of the RaCInG vs. CODEFACS derived kernel values (STAD + SKCM TCGA analysis; 71,442 kernel values). See also Figure S2.

We assessed the robustness of RaCInG to a maximum 10% variation in the cellular composition (STAR methods). When varying the tumor cell composition for the TCGA stomach adenocarcinoma (STAD) patients, we observed an almost perfect agreement between the kernel values obtained using the original cellular composition and perturbed conditions (Figure 2A), with a maximum relative error in the output equal to 6.8% after an input perturbation of maximally 10%. Similar results were obtained when perturbing different cell types and/or using different datasets (Figure S2A).

To assess alignment between Monte Carlo and kernel fingerprints, we rely on the law of large numbers based on the results from our accompanying mathematical paper.^20^ Convergence is expected when the least abundant cell type in a feature (e.g., given cell types A, B, and C for the wedge *W_A_B_C*, the one with lowest quantification based on the C-matrix) has C-matrix value larger than 1/N (where N is the number of cells in the simulation). Features satisfying this property were called *stable*. Features with all cell types even bigger than $1/\sqrt{N}$ were called *super-stable* and are expected to show even better convergence than stable features. On the contrary, features containing at least one cell type for which the C-matrix entry was smaller than 1/N, were called *unstable*.

For each patient, we performed 100 Monte Carlo simulations with N=10,000 for the STAD TCGA cohort and compared the derived features with those of the kernel method. Super-stable features showed excellent alignment (Pearson r = 0.999), the stable features had good correlation (Pearson r = 0.959), while the unstable features strongly deviated (Pearson r = −0.033; Figure 2B). Similar results were observed for other datasets (Figure S2B).

Overall, RaCInG demonstrated stability to small variation in the C-matrix and alignment between Monte Carlo and kernel values for sufficiently large N. Moreover, in case we cannot use the kernel method, the concept of (super-)stability helps to determine a suitable N for the Monte Carlo method based on the C-matrix.

### RaCInG is robust to prior knowledge on expressed ligands and receptors

To assess the impact of variations in the L- and R-matrices, we compared graphs obtained using our general L- and R-matrices to those based on patient-specific matrices derived using "confident deconvolution for all cell subsets" (CODEFACS),^7^ a method to deconvolve bulk transcriptome given the corresponding cell fractions. Applying a threshold on cell type-specific gene expression, we identified ligands and receptors expressed in each sample (STAR methods).

The kernel values from general and patient-specific matrices showed good agreement across cell type pairs (Figure 2C) (Pearson r correlation > 0.4 and *p* < 0.001). The natural killer (NK) cells, which are the most unstable (i.e., lowest abundance) and the most uncertain (i.e., low agreement between deconvolution methods, Figure S3), showed the weakest correlation.

Fitting a linear regression model (cross-validation with 1,000-folds of 20 randomly selected patients), we observed a linear relationship between the kernels values from general and CODEFACS-derived matrices (Figure 2D; mean squared error = 0.068; r^2^ = 0.48).

Overall, this analysis shows that RaCInG networks are very consistent when using general or patient-specific L- and R-matrices. Moreover, they confirm that the definition of unstable, stable, and super-stable cell types provide very useful information on the reliability of the resulting kernels.

### RaCInG is consistent with state-of-the-art methods

We validated RaCInG networks by comparing them with those derived from "ligand-receptor interactions between cell subsets" (LIRICS),^7^ a state-of-the-art method for inferring CCIs from bulk RNA-seq data, and to spatial transcriptomics data.

**Figure 3:**
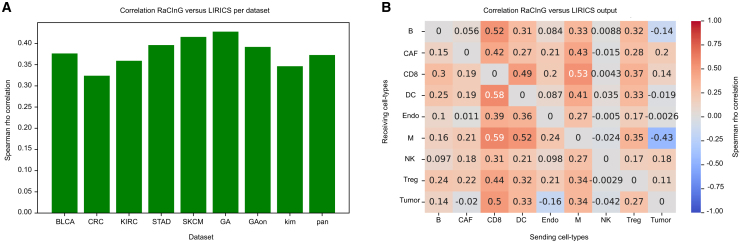
Agreement between LIRICS and RaCInG CCI networks (A) Global Spearman rho correlation (across all patients) between the RaCInG kernel values and the CCI derived from the LIRICS cell type-specific ligand-receptor pairs (81 kernel comparisons per patient in each dataset). (B) Spearman rho correlation between RaCInG and LIRICS for given cell types (2,322 kernel comparisons per cell pair; color bar indicates Spearman rho correlation).

Starting from curated ligand-receptor pairs, LIRICS defines “active” or “inactive” interactions in a specific sample based on the deconvoluted cell type-specific transcriptome obtained with CODEFACS. To compare with RaCInG, we computed quantitative scores for each cell-cell pair by counting the “active” interactions. Across patient cohorts, we observed a significant positive correlation between the RaCInG and the LIRICS CCI networks (Figure 3A) (Spearman rho range, 0.32–0.43; *p* < 0.0001). Differences between the two methods, in terms of prior knowledge and problem formulation, explain some inconsistencies (Figure 3B). For example, LIRICS does not account for interactions between cells of the same type, while RaCInG does, justifying zero correlations on the diagonal.

We observed high concordance (Spearman rho > 0.4) (Figure 3B) between the methods, when examining interactions among (super)stable cell types known to intricately shape the TME. Specifically, all pairwise interactions involving dendritic cells (DCs), CD8^+^ T cells (CD8), and macrophages (M), which are pivotal for orchestrating the antitumor immune response within the tumor milieu (DC⇄CD8, M⇄CD8, CD8⇄DC), exhibited robust agreement. This finding underscores the importance of these interactions in fine-tuning immune responses against the tumor.^22^ Similarly, we observed good agreement for B cells regulation of CD8, which take place in tertiary lymphoid structures (TLSs). TLSs have been shown to often correlate with prognosis and clinical outcome upon immunotherapy.^23^ Other examples of strong agreement are critical immunoinhibitory pathways, such as the regulatory influence of cancer-associated fibroblasts (CAFs) on CD8 and Ms (CAF→CD8, CAF→M), as well as the regulatory T cells (Tregs) and tumor cells' modulation of CD8 activity (Treg→CD8, tumor→CD8).

Conversely, weaker correlations occurred for NK cells, particularly when they functioned as receivers (Figure 3B). This finding aligns with the inherent challenges in reliably assessing cell types with lower abundance, such as NK cells (Figure S3), as previously highlighted also in the LIRICS paper.^7^ Tumor cells also showed weaker correlations, except for tumor→CD8 (Figure 3B). This discrepancy reflects differences in method assumptions, as LIRICS applies binary thresholds that fix the number of active interactions per sample, potentially leading to compensatory mismatches.

To further validate the CCIs inferred by RaCInG, we used a dataset with matched bulk RNA-seq and spatial transcriptomics data for four breast cancer patients.^24^ Using RaCInG on bulk data, we derived kernel values encoding CCIs, while deconvolution on the spatial data provided a quantification of cellular composition for each spot of the slide (STAR methods). Although RaCInG is a non-spatial model, we hypothesized that cell pairs with a high kernel value might also lie closely together spatially, making it easier for them to communicate. Indeed, kernel values correlated positively with co-localization scores (Spearman rho = 0.46; *p* < 0.001) (STAR methods), showing alignment between RaCInG and spatial data despite different methodologies.

Overall, RaCInG showed a good agreement with LIRICS, both at the global level (Figure 3A) and for the most important cell-cell pairs (Figure 3B). Moreover, RaCInG can extend beyond the analysis of LIRICS by capturing same-cell type interactions, communication clusters (e.g., triangles) and global network features (e.g., the GSCC). Additionally, RaCInG-inferred CCIs were validated using spatial data, suggesting that highly communicating cell types tend to be more spatially clustered and vice versa.

### Network features correlate with immune response and microenvironment subtypes

Having established the robustness and reliability of RaCInG, we explored how RaCInG can be used to infer information about the TME. We first correlated the direct communication, wedge, and triangle fingerprints individually to the immune response score, computed using EaSIeR^25^ (STAR methods), for 8,562 TCGA patients from 18 solid cancers.

**Figure 4:**
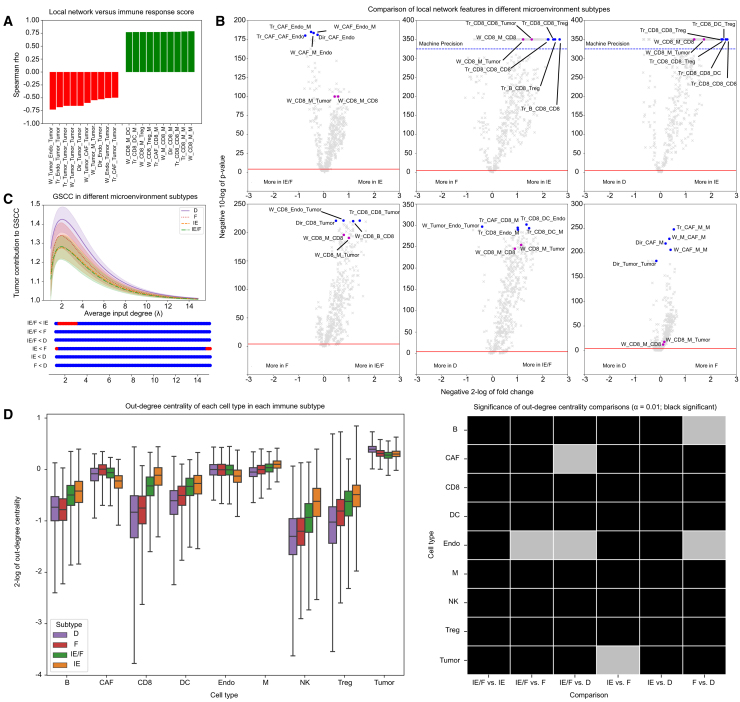
RaCInG results on TCGA datasets (A) Spearman rho correlation between RaCInG features and the immune response score across 8,561 TCGA patients. Only the 10 most strongly correlated features are shown. (B) Volcano plots comparing network-based features identified by RaCInG in pairwise comparisons of microenvironment subtypes across cancer types, with significance threshold (red line, α=0.05 after Bonferroni correction) and machine precision (blue dashed line). The x axis shows the fold change between the average feature values for each group, and the y axis the negative 10-log of the Wilcoxon rank-sum test’s *p* value. The five most significant features are highlighted in blue, and interactions W_CD8_M_Tumor and W_CD8_M_CD8 are highlighted in purple for all comparisons. (C) Size of the GSCC in TME subtypes for various input values of λ. Below the plot, significant differences between groups are indicated by blue (significant) or red (non-significant) dots (one-sided Mann-Whitney U test at significance level α=0.05). Comparisons with no significant results have been omitted. The shaded area represents the interquartile range (Q1–Q3). (D) The distribution of out-degree centrality of all patients in each immune subtype (left). Outliers in whiskers beyond three times the interquartile range have been omitted (see Figure S4B for outliers). Significance of each boxplot comparison via a two sided Mann-Whitney U test (α=0.05) after Bonferroni correction is given on the right (black comparisons are significant). See also Figure S4.

We observed that, out of the 615 local features (direct communication, wedges, triangles), 288 (47%) strongly correlated with immune response (absolute Spearman rho > 0.5; *p* < 0.01 after Bonferroni correction) (Figure 4A). Among these features, wedges and triangles often had higher correlations than direct communication features that formed a subset of them (e.g., *W_Tumor_Endo_Tumor* vs. *Dir_Endo_Tumor*; rho = −0.726 vs. −0.526; *p* < 0.001). Wedges and triangles can be more informative than simple features, as they describe intercellular communication clusters that give more detailed insight into the intercellular mechanisms driving cancer development.^22^^,^^26^

Fingerprints consisting of tumor cells, endothelial cells (Endo), CAFs, and absence of immune cells had a strong negative correlation with immune response. Conversely, those involving immune cells, especially CD8 and Ms, had strong positive correlation, consistent with the known link between immune response and immune cell infiltration.^22^^,^^27^ CD8, as a key players in recognizing and eliminating cancer cells, were central to these immune response-associated fingerprints.^27^ Interestingly, one feature involving M communication with tumor cells had a strong negative correlation with the immune response score (*W_Tumor_M_Tumor*; rho = −0.545; *p* < 0.001), suggesting tumor cells may attempt to repolarize Ms toward a pro-tumor phenotype rather than Ms exhibiting this role outright. In tumors with weak immune response, the main communication players were tumor cells, CAFs, and Endo.

We further used RaCInG to compare CCI networks in different microenvironment subtypes. We considered the four subtypes defined in literature^28^: immune enriched (IE), IE-fibrotic (IE/F), F, and immune deprived (D). While both the IE and IE/F subtypes are enriched in immune cells, the IE subtype presents a more immune active microenvironment than the IE/F, which is instead characterized by higher CAF activation. The D subtype is instead characterized by a higher content of malignant cells and has little immune or stromal cell activation. IE and F tumors were shown to have positive and negative correlation with response to ICB therapy respectively.

Also in this context, we observed that Ms can have a dual role depending on their cellular communication (Figure 4B). Ms working in conjunction with CD8 characterized IE environments (see, e.g., Tr_CAF_CD8_M and Tr_CD8_DC_M appearing in the top five interactions in the IE/F vs. D comparison, or W_CD8_M_Tumor and W_CD8_M_CD8 appearing with lowest possible *p* value in the IE vs. F and IE vs. D comparison). These observations are in agreement with a higher proportion of anti-tumor Ms that have been observed in the IE tumors.^28^

Interestingly, triplets involving CAFs, Ms and Endo (e.g., *Tr_CAF_Endo_M* in the IE vs. IE/F comparison; *p* < 0.001 and fold-change 0.71) differentiate the more hostile microenvironment subtype IE/F from the more favorable IE subtype. This is in line with a recent report that such triplets lead the communication in human breast cancer TME.^26^

When looking at the GSCC (Figure 4C), we observed that IE environments have lower tumor cell contribution, as communication is dominated by immune cells, Endo, and CAFs. Unlike local network features (i.e., communication between pairs or triplets of cells), the computation of the GSCC depend on the chosen average degree in the network (λ), although it tends to be consistent for a wide range of λ.

Consistently, when considering out-degree centrality the influence of tumor cells on the TME diminishes as the microenvironment subtype changes from D to IE (see e.g., the significant decrease in CAF and tumor influence) (Figure 4D), while the influence of immune cells increases (see, e.g., the influence of CD8). Similar observations can also be made from in-degree centrality (Figure S4A).

Overall, we have shown that network features are associated with immune response and microenvironment subtypes, and that preferential cellular interactions can explain the dual role that certain cell types, such as Ms, can exhibit in the microenvironment.

### Network features can predict response to ICB

As the graph features derived by RaCInG provided mechanistic understanding in terms of CCIs about patients’ immune phenotype, we extended the analysis into investigating patients’ response to anti-PD1 immunotherapy^29^^,^^30^^,^^31^ (STAR methods).

**Figure 5:**
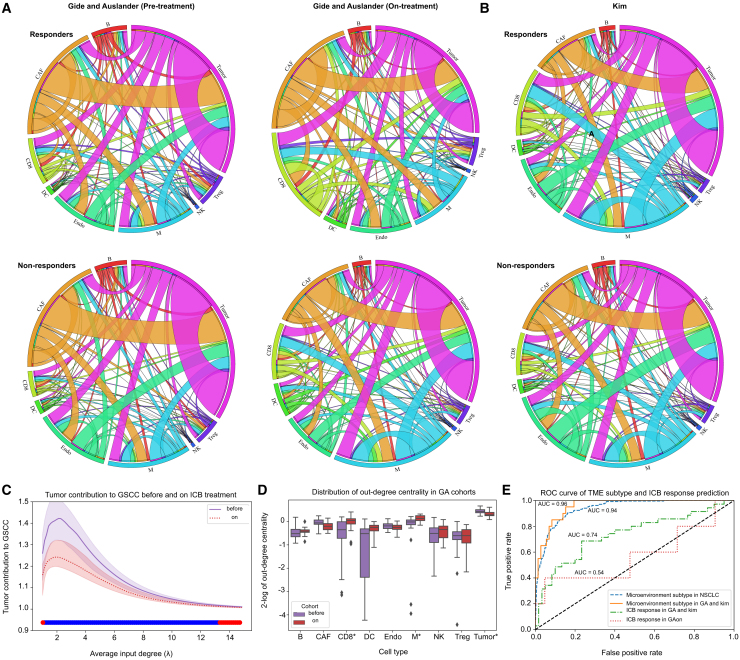
Effect of ICB on TME analyzed through RaCInG (A) Circos plot of average kernel values in responder and non-responder groups of the Gide-Auslander cohorts. The size of each ribbon indicates the fraction of total communication each cell-type is part of. The thickness of the lines in between two cell-types indicates how much these cell types communicate. (B) Circos plots for the Kim cohort. (C) Effect of ICB on the tumor contribution in the largest strongly connected component in the TME (Gide-Auslander cohorts), with significant differences indicated by blue below the plot (one sided Mann-Whitney U test, α=0.05)The shaded area represents the interquartile range (Q1–Q3). (D) Distribution of out-degree centrality of all patients in the Gide and Auslander cohorts. Significance of boxplot comparisons (two-sided Mann-Whitney U test; α = 0.05) is indicated with a star at the cell's name (see also Figures S4B–S4G). (E) Receiver operating characteristic (ROC) curve for prediction of ICB response using RaCInG kernel values with models trained on TCGA microenvironment subtype data. See also Figures S4 and S5.

First, we analyzed two melanoma datasets (Gide-Aulander cohorts^29^^,^^30^) with known ICB response and RNA-seq data from samples collected before (*n* = 50) and on (*n* = 26) treatment. We computed the average number of arcs between two given cell types based on the *unnormalized* kernel for the responder and non-responder groups, and used it as a measure of direct communication between cell types in the TME (Figure 5A; STAR methods). We compared pre- vs. on-treatment samples to study the effect of ICB therapy on the CCI network. For the responding patients we observed a large increase of CD8 communication after treatment (sum of arc averages 0.1453 vs. 0.28748 for before and on-treatment samples, respectively). Specifically, we saw that both the amount of communication from tumor to CD8 (arc average 0.0292 before treatment vs. 0.0450 on treatment, respectively) and from Ms to CD8 (0.0155 vs. 0.0317, respectively) doubled. Moreover, the amount of communication in between CD8 almost quadrupled (0.0115 vs. 0.0400, respectively), suggesting a pivotal role of T cell-T cell communication for controlling T cell activity for an effective response to ICB.^32^ For the non-responder patients in the same cohort, we still saw an increase in CD8 communication (the sum of all expected arc counts involving CD8 was 0.0817 before treatment vs. 0.175 on treatment), but not in tumor communication (0.5588 vs. 0.5223) upon ICB treatment. We also noted that M communication to CD8 tripled (0.0092 vs. 0.0339).

The positive association between ICB response and Ms to CD8 communication in the pre-treatment samples (i.e., higher arc count for responders, 0.0155 vs. 0.0092) could be justified by a significantly higher relative abundance of M1 (anti-tumor) to M2 (pro-tumor) polarized Ms in the responders (Wilcoxon rank-sum test; *p* = 0.018 comparing M2/M1 ratio in responders vs. non responders) (Figure S5D). In fact, M1-like (classically activated) Ms are known to positively regulate CD8 through secretion of pro-inflammatory cytokine and chemokines and efficient antigen presentation creating a more inflamed environment that could be beneficial for ICB response.^33^^,^^34^^,^^35^^,^^36^

On the contrary, we did not observe any significant difference in M2/M1 ration between responders and non-responders on treatment (*p* = 0.79) (Figure S5D). Therefore, we hypothesized that the higher increase in pro-tumor Ms activity observed after treatment in non-responders could possibly be due to M-mediated suppression of the anti-tumor activity of CD8, causing resistance to ICB treatment.

To further characterize the role of Ms to CD8 communication, we computed the ligand-receptor interaction probabilities driving this interaction in the before treatment samples (Figure S5A; STAR methods). We observed that both chemokine (C-X-C motif) ligand 3 and 7 (CXCL3 and PPBP) had more than double the probability to interact with interleukin 8 receptor beta (CXCR2) in the non-responder group compared to the responder group. Although not well characterized specifically for Ms to CD8 interactions, these LR pairs are in general linked to immunosuppression^37^^,^^38^ and tumor progression,^38^^,^^39^ in line with the observed pro-tumor effect. Also, the interaction between human leukocyte antigen (HLA) class I histocompatibility antigen, alpha chain E (HLA-E), with killer cell lectin-like receptor K1 (KLRK1) had more than double the probability in non-responders. This can be explained by (non-classical) HLA-E interactions being identified as an immune checkpoint,^40^^,^^41^ although the specific role of KLRK1 remains unclear. Finally, we observed that communication between clusters of differentiation 48 and 244 (CD48 and CD244) was large in general, but much larger in non-responders specifically. This can be explained by the fact that CD244 promotes T cell exhaustion through CD48.^42^ All in all, these findings suggest more pro-tumor interactions of Ms in the TME for non-responders to ICB.

Next, we analyzed responder and non-responder gastric cancer patients treated with anti-PD1 (Kim cohort, *n* = 45) (Figure 5B),^31^ observing that Ms communicated more with CD8 in the responder (arc count of 0.0446) than in the non-responder group (arc count of 0.0245). Similarly to our observations in the Gide-Auslander cohort, this could be explained by a significantly lower M2/M1 ratio (*p* = 0.039) (Figure S5D), also backed by the underlying ligand LR interactions (Figure S5B). Additionally, the top LR interactions for NR are more highly expressed in tumor compared to normal (non-tumor) samples in matched STAD TCGA samples (Figure S5C). Finally, we observed that B cells were in general more active in non-responders (sum of arc counts involving B cells is 0.0521 in responders vs. 0.0758 in non-responders). A possible explanation to this behavior is the formation of regulatory B cells. This phenotype of B cells play a role in tumor progression and immune system suppression in gastric cancer.^43^

We subsequently assessed whether tumor communication is reduced after applying anti-programmed cell death 1 (PD-1) therapy. By calculating the tumor contribution to the largest connected component in the before- and on-treatment datasets, we showed that the on-treatment group indeed had generally lower tumor activity (Figure 5C), explainable by restored immune response in the tumor by blocking PD-1.^44^ This observation was not as consistent as differences in immune phenotypes (Figure 4B), due to the smaller sample size. There was also no significant difference when spitting up the two datasets into responders and non-responders (Figure S5E).

Consistently, we observed a significant increase in CD8 influence in patients on ICB blockers, while tumor influence decreased (Figure 5D). Also, M influence significantly increased, further supporting the alleged dual role of Ms. In-degree centrality shows similar results (Figure S4C).

Finally, we assessed the predictive value of RaCInG fingerprints by training a logistic regression model on the four microenvironment subtypes using the STAD and SKCM TCGA datasets. We observed that kernel values were good predictors of microenvironment subtypes when testing on a different TCGA cancer type (NSCLC; area under the curve [AUC] = 0.94) as well as on the validation datasets (Gide, Auslander, and Kim cohorts, AUC = 0.96) (Figure 5E). Subsequently we applied the model to predict response to anti-PD-1 therapy in the three validation datasets. In line with correlations between immune phenotypes and response,^28^ IE prediction was mapped to responders, while other predictions were mapped to non-responders. When using the model to predict ICB response using before-treatment data we saw that RaCInG performs relatively well (AUC = 0.74), considering that the IE subtype does not have a one-to-one correspondence with ICB response. Interestingly, the model performance decreased in the on-treatment cohort (AUC = 0.57), likely given by the dynamic evolution of the TME as changes in the TME subtyping were previously observed in responders on treatment.^28^

Overall, we have shown that network features derived using RaCInG are predictors of immune phenotype and ICB response and that they are informative on the underlying mechanisms characterizing (lack of) response to treatment. In additional experiments we have also shown that inclusion of triangles and wedges is most helpful in small datasets, and that the normalization in general improved the predictions (see Figures S5F and S5G).

## Discussion

RaCInG is a new computational tool specifically developed to construct patient-specific CCI models using bulk RNA-seq, which are widely available and clinically relevant. This fills a critical gap, as few methods exist for analyzing CCIs from bulk data. Leveraging random graph theory, RaCInG provides a modular pipeline to build and analyze CCIs at the level of individual cells. Its kernel method offers theoretical assurances about network accuracy,^20^^,^^21^ allowing efficient extraction of information on the patient’s TME. Where these assurances are unavailable, the Monte Carlo method serves as a robust alternative, and we have demonstrated their consistency and complementarity. Importantly, RaCInG can mitigate the bias introduced by cell-type quantification, thereby equalizing all network features. This normalization also enhances predictive accuracy in specific case studies.

RaCInG represents an advancement over existing methods by reconstructing CCIs in the TME at the resolution of individual cells by incorporating stochasticity. By analyzing an ensemble of admissible networks consistent with input data, RaCInG surpasses traditional methods reliant on mean field approximations, enabling the identification of robust network features like the GSCC and degree centrality, which give further insights in the TME.

RaCInG networks can also be adapted to commonly used direct cell-type-level interactions. We have shown consistency between RaCInG and LIRICS, the state-of-the-art methods for CCI inference from bulk data, and with spatial transcriptomics data. Discrepancies between RaCInG and LIRICS could be explained by intrinsic methodological differences. Similar disagreement has also been observed in single-cell methods^45^ and would support the use of ensemble methods when interested only in cell-type communication analysis.

Beyond direct cell communication, RaCInG can consider features involving more than two cell types (e.g., wedges and triangles), which have emerged as drivers of cancer progression.^22^^,^^26^ We have successfully replicated key triplets (e.g., *Tr_CAF_Endo_M)* previously identified from single-cell data analysis. We found these triplets are often better associated with tumor characterization than the corresponding subsets of direct communication pairs. Our results highlight the importance of studying CCI networks comprehensively. RaCInG further exemplifies this by analyzing graph features unrelated to direct communication. For instance, cell contribution to the GSCC and degree centrality of different cell types provides insights into immune phenotypes and immunotherapy efficacy, highlighting the broader utility of network-level features.

RaCInG can provide functional insights into the TME by revealing how CCIs regulate cellular phenotypes, possibly explaining the dual role that certain cell types can have in different contexts.^27^^,^^46^ For example, Ms can be associated with an anti-tumor or a pro-tumor TME subtype, depending on whether they preferably interact with CD8 or with CAFs, respectively.

We further used RaCInG to analyze TME changes after ICB therapy, finding that pre-treatment communication between Ms and CD8 is linked to better ICB response in tumors enriched with M1-like Ms. In contrast, non-responders showed more pro-tumor LR interactions and a higher increase in M to CD8 communication during treatment. Combined with the observed relative increase in M2-like Ms, this suggests a shift toward more immunoinhibitory interactions in non-responders. If this is the case, combining anti-PD1 therapy with M-targeting treatment could be a better treatment strategy.^33^^,^^47^ These findings underscore M plasticity and highlight the critical role of M-CD8 interactions in determining ICB efficacy, consistent with a recent study^48^ illustrating how CCIs extend our understanding of TME polarization beyond quantification.

Our TCGA case study primarily focused on undirected graph features, despite the model’s ability to handle directed features. This simplification comes from the observation that directed features involving the same cell types often had similar relevance and reflects the bidirectional nature of ligand-receptor interactions, where both the ligand and receptor cell types react to the interaction, partially obscuring its directionality.^10^

Although we demonstrated RaCInG’s potential through various case studies, its flexibility and modularity allow adaptation to diverse research needs. This includes integrating additional network fingerprints (e.g., intricate graphlets like stars) and accommodating different input data sources, such as directly measured cell quantification (e.g., by flow cytometry) or context-specific information on ligand-receptor expression (e.g., by transcriptome deconvolution as exemplified in this paper using CODEFACS). Looking ahead, expanding random graph methods to include geometry^49^ could harness spatial data availability, extending RaCInG’s applicability to spatial transcriptomics^50^ or immunohistochemistry.^51^

In conclusion, RaCInG stands poised as a versatile tool for investigating how CCIs shape tissue phenotypes across diverse contexts. We envision RaCInG application to investigate intercellular communication in different physiological (e.g., cell development^52^^,^^53^^,^^54^^,^^55^ or tissue homeostasis^56^) and pathological contexts.^57^

### Limitations of the study

RaCInG is less reliable for cell types with low C-matrix values, such as NK cells. These cells cannot be accurately estimated with current deconvolution methods due to their transcriptional similarity with T cells. Their low quantification makes features involving NK cells unstable when using Monte Carlo simulations, introducing noise to the analysis. To address this, RaCInG provides guidelines on the reliability of Monte Carlo features, distinguishing between (super)stable and unstable cell types.

While RaCInG could, in principle, be applied to single-cell RNA-seq data, we did not test it on this data type due to the availability of other methods specifically tailored for single-cell analysis. Conversely, the current implementation of RaCInG does not work with spatial transcriptomics data. However, the underlying random graph theory provides a solid foundation to incorporate geometry, suggesting that RaCInG could be extended in the future to analyze spatial data.

Additionally, while RaCInG effectively infers higher-order interactions (e.g., wedges and triangles), interpreting these features remains challenging. Although our findings highlight their relevance, further efforts are needed to understand the functional roles of these complex network structures.

Last, the emphasis on undirected graph features in our case studies reflects a simplification of the model rather than a limitation of RaCInG itself. Directed features remain underexplored but could reveal new insights into direction-specific effects, particularly for ligand-receptor interactions.

## Resource availability

### Lead contact

Further information and requests for resources should be directed to and will be fulfilled by the Lead contact, Federica Eduati (f.eduati@tue.nl).

### Materials availability

This study did not generate new unique reagents.

### Data and code availability

• This paper analyzes existing, publicly available data. These accession numbers for the datasets are listed in the key resources table.
• All original code has been deposited at https://github.com/SysBioOncology/RaCInG and is publicly available. Repositories of other methods are listed in the key resources table.
• Any additional information required to reanalyze the data reported in this paper is available from the lead contact upon request.

## Acknowledgments

The authors acknowledge the support of the Immunoengineering Program of the Institute for Complex Molecular System. F.E. was supported by the Netherlands Organization for Scientific Research (NWO) Gravitation programme IMAGINE! (project number 24.005.009). F.F. was supported by the 10.13039/501100002428Austrian Science Fund (FWF) (no. T 974-B30 and FG 2500-B) and by the Oesterreichische Nationalbank (OeNB) (no. 18496). The computational results presented here have been achieved in part using the LEO HPC infrastructure of the University of Innsbruck. The results shown here are in part based on data generated by TCGA Research Network (https://cancergenome.nih.gov). We would like to thank Livy Nijhuis for testing the code. We would like to thank the developers of CODEFACS and LIRICS, and in particular Dr. Kun Wang, for sharing their code to perform comparative analysis. We thank the reviewers for very useful comments and suggestions.

## Author contributions

P.v.d.H. and F.E. designed the research. M.v.S., O.L.S., P.v.d.H. and F.E. discussed how to use random graph models in biological context. M.v.S. defined and implemented the mathematical formulation of the model under the supervision of P.v.d.H. O.L.S. analyzed the data used for the case study and transformed it into input matrices for the mathematical model under the supervision of F.E. M.Z. and C.Z. analyzed the spatial transcriptomics data. F.F. devised the approach for cell-type quantification using an ensemble of deconvolution algorithms and supervised the analysis of spatial transcriptomics data. M.v.S., O.L.S., P.v.d.H., and F.E. contributed to the interpretation of the results. M.v.S. and F.E. co-wrote the manuscript with input from all authors. All authors discussed the results and commented on the manuscript.

## Declaration of interests

The authors declare no competing interests.

## Declaration of generative AI and AI-assisted technologies in the writing process

During the preparation of this work the authors used ChatGPT in order to improve readability and shorten some portions of the text. After using this tool, the authors reviewed and edited the content as needed and take full responsibility for the content of the published article.

## STAR★Methods

### Key resources table

**Table:** 

REAGENT or RESOURCE	SOURCE	IDENTIFIER
**Datasets**		

Gene expression data for 18 solid tumors (BLCA, BRCA, CESC, CRC, GBM, HNSC, NSCLC, KIRC, KIRP, LIHC, LUAD, OV, PAAD, PRAD, SKCM, STAD, THCA and UCEC).	BROAD Institute	https://gdac.broadinstitute.org
Gene expression data for Gide cohort.	Gide et al.^29^	ENA: PRJNA476140
Gene expression data for Auslander cohort.	Auslander et al.^30^	ENA: PRJEB23709
Gene expression data for Kim cohort.	Kim et al.^31^	ENA: PRJEB25780
Gene expression data for Wu cohort.	Wu et al.^24^	GEO: GSE176078

**Software and algorithms**		

R Project for Statistical Computing	The R Foundation	http://www.r-project.org/; RRID: SCR_001905
Python programming language	Python Software Foundation	http://www.python.org/; RRID: SCR_008394
RaCInG	This manuscript	https://github.com/SysBioOncology/RaCInGhttps://doi.org/10.5281/zenodo.14717719
CODEFACS and LIRICS	Wang et al.^7^	https://zenodo.org/record/5790343
immunedeconv R package v2.1.0	Sturm et al.^17^	https://github.com/omnideconv/immunedeconv; RRID: SCR_023869
LIANA R package v0.1.10	Dimitrov et al.^45^	https://github.com/saezlab/liana
OmnipathR R package v3.7.0	Türei et al.^18^	https://github.com/saezlab/OmnipathR
easier	Lapuente-Santana et al.^25^	https://bioconductor.org/packages/release/bioc/html/easier.html
spacedeconv	Zackl et al.^58^	https://github.com/omnideconv/spacedeconv

**Other**		

Normalized kernel values	This paper	Table S1
Unnormalized kernel values	This paper	Table S2
Fingerprints computed by RaCInG (kernel method)	This paper	Table S3
Spatial colocalization scores	This paper	Table S4
Fingerprints computed by RaCInG (Monte-Carlo method)	This paper	Table S5

### Method details

#### Cancer data acquisition and transformation

In the context of modeling the TME, RaCInG requires different types of biological information. We first annotated which ligand-receptors are specific for the different cell-types of interest by leveraging curated literature resources^18^ and cell-type specific RNA-seq data.^59^ And then, we used bulk RNA-seq data to quantify cell type fractions and ligand-receptor bindings for each individual patient.

To better characterize the cell-cell communication network produced by RaCInG, we gathered information about the TME subtype of patients (from literature) and their anti-cancer immune response (inferred from bulk RNA-seq).

#### Bulk RNA-sequencing data

##### The Cancer Genome Atlas (TCGA)

Gene expression data for 18 solid tumors: BLCA, BRCA, CESC, CRC, GBM, HNSC, NSCLC, KIRC, KIRP, LIHC, LUAD, OV, PAAD, PRAD, SKCM, STAD, THCA and UCEC were downloaded via the Firehose tool from the BROAD Institute (https://gdac.broadinstitute.org), released January 28, 2016. We selected primary tumor or metastatic (only in the case of melanoma) samples, resulting in a total of 8562 patients. We additionally considered the 67 STAD normal (non-tumor) samples with matched tumor samples.

We extracted the gene expression data from “illuminahiseq_rnaseqv2-RSEM_genes” files. From these data, we used “raw_count” values as counts, and we calculated transcripts per million (TPM) from “scaled_estimate” values multiplied by 1,000,000. We first removed those genes with a non-valid HGNC symbol and then we averaged the expression of those genes with identical HGNC symbols.

##### Data of patients treated with immunotherapy

Gene expression data for melanoma (Gide^29^ and Auslander^30^ cohort), gastric cancer (Kim^31^ cohort) and breast cancer (Wu^24^ cohort) was available from published datasets of patients treated with anti-PD1 therapy, which also include information about patients’ best overall response (Table S6 for more details).

For each cohort, we downloaded FASTQ files of RNA-seq reads from the Sequence Read Archive (SRA, https://www.ncbi.nlm.nih.gov/sra/). We used quanTIseq to process the data.^60^ First, Trimmomatic^61^ is used to remove adapter sequences and read ends with Phred quality scores lower than 20, discard reads shorter than 36 bp, and trim long reads to a maximum length of 50 bp (quanTIseq preprocessing module). Then, Kallisto^62^ is applied on the pre-processed RNA-seq reads to generate gene counts and TPM using the “hg19_M_rCRS” human reference (quanTIseq gene-expression quantification module).

##### Spatial transcriptomics

Spatial deconvolution analysis was performed using the R package spacedeconv^58^ (https://github.com/omnideconv/spacedeconv) to infer cell spot composition using cell2location. The single-cell RNA-seq reference dataset employed was the one provided by Wu et al.,^24^ which comprised 100,064 cells from 26 primary tumors of three major clinical subtypes of breast cancer, including 11 ER+, 5 HER2+, and 10 TNBC. The dataset was preprocessed using Seurat 4.3.0, wherein cycling cells were eliminated, and the dataset was further subsetted using the subsetSCE() function of spacedeconv, with the ncells parameter set to 5000 and the scenario parameter set to "even" to reduce extensive computational resources. The “minor” cell type annotation from the metadata was used for the deconvolution analysis. The spatial transcriptomics data was obtained from the same study^24^ (slides from "patient_4290″, "patient_4465″, "patient_4535″, and "patient_44971″). Both the single-cell reference dataset and the spatial slide were further preprocessed with the `preprocess()` function of the spacedeconv package with default settings, removing observations (cells or spots) with a unique molecular identifier (UMI) count below 500 and eliminating genes with zero counts across all observations. Cell2location was run with unnormalized counts for both the spatial and single-cell reference data. Cell2location operates with a two-step approach, including scRNA-seq informed signature building, followed by deconvolution of the spatial transcriptomics object. To build the signature we used the `build_model` function of spacedeconv with the following settings, epochs = 250 (number of epochs to train the cell2location model), gpu = TRUE (whether to train on GPU), and assay_sc = "counts" (assay to extract from the reference single-cell object analyzed). For the second step, we used the `deconvolute` function of spacedeconv with the following parameters: epochs = 30000, gpu = TRUE, assay_sp = "counts" (assay to extract from the spatial transcriptomics object analyzed).

From the cellular deconvolution values per spot, we derived a colocalization score per cell type. This is done by first adding the deconvolved values from cell types of a similar class in the bulk dataset (see Table S7 for the mapping), and then multiplying this combined score for two given cells together. Finally the resulting values get added over all spots and over each patient divided by the total number of spots to derive the colocalization score per cell-pair that is used in RaCInG. These were the values compared to the RaCInG kernels derived from bulk RNA-seq of the same dataset.

##### TME subtypes

We used a previously defined classification of the TME to assign patients into different subtypes: Immune-Enriched Fibrotic (IE/F), Immune-Enriched Non-Fibrotic (IE), Fibrotic (F) and Desert (D).^28^ The TME subtype associated with each patient was provided by the original work for TCGA datasets as well as for Gide-Auslander cohorts.

#### Transformation of RNA-seq into RaCInG input

##### C-matrix generation

We used in silico deconvolution^63^ to estimate cell fractions from bulk-tumor RNA-seq data. To obtain robust cell fraction estimates, we used a consensus approach based on six, well-validated deconvolution methods accessible through the immunedeconv^17^ R package v2.1.0: quanTIseq,^60^ EPIC,^64^ ConsensusTME,^65^ xCell,^66^ TIMER,^67^ and MCP-counter.^68^ Of note, we based our strategy on a selection of *first-generation* deconvolution methods,^69^ i.e., methods based on pre-computed cell-type transcriptional signatures, which cover the major immune and non-immune cell types in the TME and have been extensively characterized and benchmarked in previous studies in terms of strengths and limitations.^17^^,^^70^ We did not consider second-generation methods that can be trained using annotated single-cell RNA-seq data for the difficulty of validating their signatures and ultimate results in dependence to the input data and application context.

Our consensus strategy is centered on two main deconvolution tools, quanTIseq and EPIC, which were selected for their unique capability of estimating cell fractions referred to the overall composition of the tumor sample (not supported by any of the other methods). The remaining methods were used to confirm and/or refine the estimates as explained in the following. Practically, quanTIseq was used to estimate cell fractions for CD8^+^ T cells, B cells, Tregs, M1 and M2 macrophages, which showed a high correlation with the other deconvolution methods (Figure S3). Since M1 and M2 signatures do not recapitulate their diversity in the tumor and given the limited availability of methods to derive a consensus we decided to sum them and consider macrophages as a unique cell type. EPIC was used to estimate CAFs (absent in quanTIseq signature), NK cells (low consensus agreement for quanTIseq), and tumor cells (high agreement with quanTIseq estimates, but more accurate as they do not include endothelial and epithelial cells), and normal cells (endothelial cells). Treg and NK cell fractions that were given a null score by xCell, were set to zero. Given the low agreement of EPIC and quanTIseq on DC fractions compared to other methods, we used a three-step consensus approach: 1) we scaled in the 0–1 range DC scores obtained with xCell, MCP-counter, and TIMER; 2) we took their median; and 3) we rescale it to span the range of values covered by quanTIseq, after correction of absent cells according to xCell. Finally, cell fractions in each sample were rescaled to sum up to 1.

##### L- and R-matrix generation

Using the LIANA^45^ R package v0.1.10 and the OmnipathR R package v3.7.0, we retrieved a customized set of intercellular interactions from OmniPath,^18^ which consisted of interactions curated in the context of cell-cell communication available from six resources: CellphoneDB,^19^ CellChat,^71^ ICELLNET,^72^ connectomeDB2020,^73^ CellTalkDB^74^ and Cellinker.^75^ Then, we filtered for direct CCIs by excluding proteins related to the extracellular matrix. Additionally, protein complexes were split into individual subunits. This resulted in a total of 3081 LR interactions.

From the database of Ramilowski et al.,^59^ the gene expression of 144 human cell-types based on cap analysis of gene expression (CAGE) from the FANTOM5 project is available. We kept only the cell-types for which we could quantify their abundance based on deconvolution methods. The agreement was not perfect and certain “deconvolution” cell-types matched more than one “ramilowski” cell type, thus we aggregated them by averaging their expression because they showed high correlation between the expression of their ligands and receptors. We additionally included a pan-cancer cell type derived by using data from the Cancer Cell Line Encyclopedia (CCLE)^76^ as described in our previous study.^25^ Based on gene expression data of 583 cell lines (from 18 solid cancer types), the median expression of each gene was considered as the gene expression of the pan-cancer cell type.

Ligands and receptors were first selected based on their expression (≥10 TPM threshold) in at least one of the 10 cell-types considered, and then based on the presence of the corresponding ligand or receptor pair in the network. The 10 TPM threshold was initially used in the Ramilowski paper for the CAGE data, and it was based on known expression data from B-cells. We have previously described that this cutoff value was suitable for the CCLE RNA-seq data.^25^

The compatibility of ligand and receptors was specific for each cell type, comprising a total of 971 LR pairs.

##### L- and R-matrix generation (CODEFACS)

When CODEFACS was used to derive the L- and R-matrix we first applied the CODEFACS method proving the TPM counts from bulk RNA-seq data and the cell-type deconvolution as input. The result from this method was a patient-specific matrix that estimated for each cell-protein pair the expression (in TPM). Then, similar to the normal method to derive the L- and R-matrix, a cell-protein pair was deemed compatible if its expression exceeded the 10 TPM threshold. All in all, this resulted in a cell-ligand and cell-receptor matrix on an individual patient level rather than at a global level.

##### LR-matrix generation

Patient-specific LR pair weights were defined as the minimum of the log_2_(TPM+1) expression of the ligand and the receptor, hypothesizing that the expression of the gene at the lower level limits the LR binding affinity.

#### Computation of immune response score

We used our "easier" R/Bioconductor package^25^^,^^77^ to compute a score of immune response based on the median of the *Z* score values of 10 published transcriptomics signatures of the immune response. All these signatures were calculated according to the methodology reported by the original studies.

#### Random graph inference (Monte Carlo)

The process in which RaCInG created graphs and extracted features is independent of the application domain. Three different facets are important in this pipeline.

(1) Generation of nodes and arcs based on input data.
(2) Assignment of arcs to node-pairs.
(3) Feature extraction.

##### Generating nodes and arcs

An overview of the variables and distributions used for the random graph model is presented in Table 2. These variables correspond to (elements of) the input matrices in Figure 1.

**Table 2:** Commonly used mathematical notation in the description of RaCInG and its procedures

Symbol	Type	Interpretation	Notes
N	number	number of cells in one network instance	–
λ	number	average number of interactions per cell	–
Q	probability distribution	probability of cells having a given type, i.e., the cell-type quantification in the C-matrix of Figure 1	$q_{k}$ is the probability of one cell having type k.
P	probability distribution	probability of an interaction consisting of a given ligand and receptor, i.e., the ligand-receptor quantification in the LR-matrix of Figure 1	$p_{ij}$ is the probability of one interaction consisting of ligand i and receptor j.
L	matrix	compatibility of specific cell-types with specific ligands.	L(k,i) is the indicator that cell-type k can secrete ligand i.
R	matrix	compatibility of specific cell-types with specific receptors	R(k,i) is the indicator that cell-type k can secrete receptor j.
κ	matrix	kernel of a specific patient obtained through the kernel method	κ(A,B) is the kernel value from cell type A to cell type B.

To create the nodes for one instance of the network, RaCInG creates a list of length N with independent realizations from Q. In this list, entry l corresponds to the cell-type of node l. Similarly, to create the (unpaired) arcs for one instance of the network, RaCInG creates a list of length λN (rounded down) with independent realizations from P. Here, entry l of the list corresponds to a tuple that encodes both the ligand and the receptor of interaction l.

##### Pairing nodes and arcs

To pair nodes and arcs, RaCInG iterates over the list of interactions in the following way.

(1) It reads the type of the interaction’s ligand. Suppose it had type i.
(2) It highlights all nodes that have a type k such that L(k,i)=1.
(3) It chooses one of these nodes uniformly at random with replacement.
(4) It reads the type of the interaction’s receptor. Suppose it had type j.
(5) It highlights all nodes that have a type k such that R(k,j)=1.
(6) Independently of the previous choice, it chooses one of these nodes uniformly at random with replacement.

After this procedure is executed for all interaction pairs, we obtain an output network. To generate an ensemble of networks for one patient, RaCInG repeats the node/interaction procedure and pairing procedure a predetermined number of runs. Each run is generated independently from the previous runs.

After testing the Monte Carlo graph generation algorithm on the first one hundred patients of the SKCM dataset, generating ten graphs per patient, we note that it is sub-linear in its memory complexity and linear in its time complexity (Figure S6).

##### Feature extraction

###### Direct communication

Direct communication between two cell-types is measured by looping over all generated edges and counting how many are received and sent by the given two cell-types. We find the corresponding feature value by dividing this count by the total number of vertices N.

###### Wedges and triangles

For wedges and triangles the feature extraction is based on a network’s adjacency matrix A. In this matrix the entry $a_{ij}$ indicates the number of arcs from node i to node j. For each network, RaCInG outputs a list of paired arcs, which is transformed into an adjacency matrix. Features are then extracted from this matrix.

For example, for the wedges this is done by iterating over all rows in the matrix, recording the neighbors a given vertex connects to (together with the multiplicity of the connection) and then recording these neighbors’ subsequent neighbors. This yields a list of triplets of vertices that form wedges. The types of these wedges can subsequently be extracted and tallied for each combination of cell-types. Triangles counts are computed in a similar way.

Once this procedure is executed for each individual network in the ensemble, the average is computed over all the tallies. This provides the value of one feature for a given patient. The standard deviation is also recorded as a check to ensure the average expression value concentrates around the actual measured feature values from each network.

###### Largest strongly connected component

The size of the largest strongly connected component is computed through Tarjan’s algorithm.^78^ This algorithm outputs a list of vertices belonging to the all strongly connected components, and by contrasting the list with the generated cell-types we can also use it to compute the contribution of each cell-type to the largest strongly connected component.

#### Random graph inference (kernel)

The kernel method allows us to extract features from CCI networks in the setting where the number of cells tends to infinity. Since CCI networks usually consist of a huge number of cells, this “infinite cell perspective” provides a good approximation of the Monte Carlo method that sidesteps computational issues. When using this method, two facets are important.

(1) Kernel computation.
(2) Feature extraction.

##### Kernel computation

The kernel of a random graph model, which is usually denoted by κ(t,s), is a function that encodes for two cells with cell-type t and s what the probability of an arc appearing between them is. For RaCInG, the kernel of the model is based on all inputs in Table 2, and given by

$$\kappa \left(t,s\right)=\lambda \sum_{i=1}^{\infty }\sum_{j=1}^{\infty }\frac{p_{ij}\cdot L\left(t,i\right)R\left(s,j\right)}{\alpha_{i}\cdot \beta_{J}}\;\text{.}$$

where the values of $\alpha_{i}$ and $\beta_{j}$ are given by

$$\alpha_{i}=\sum_{k=1}^{\infty }q_{k}L\left(k,i\right)\text{,}$$

$$\beta_{j}=\sum_{k=1}^{\infty }q_{k}R\left(k,j\right)\text{.}$$

To find the kernel for a given patient, one would have to compute κ(t,s) for all cell-type pairs t and s using the input data of the given patient. Then, this kernel can be used to derive feature values. Note that in practice each sum is bounded by the amount of ligands, receptors and cell-types incorporated in the analysis, even though the theoretical sums run to infinity.

The value κ(t,s)/N can be interpreted as the probability that an interaction is generated from a fixed cell with type t toward a fixed cell with type s. For more details on the derivation and interpretation of the kernel, see our accompanying mathematical paper.^20^

##### Feature extraction

###### Direct communication, wedges and triangles

Many features like triangle, wedge and direct communication values can be derived as easy combinations of kernel values. An overview is given in the list below. Note that the kernel values and hence the fingerprints presented below are directed.

(1) Direct communication from cell type A to B: κ(A,B).
(2) Wedge communication from cell type A to B, and then from B to C: κ(A,B)·κ(B,C).
(3) Triangle communication from cell type A to B, from B to C, and then from C back to A: κ(A,B)·κ(B,C)·κ(C,A).

###### The largest strongly connected component

The largest strongly connected component in a directed graph is defined as the largest group of individual nodes such that there is a path between any two nodes and back (Figure S1B). The size of the largest strongly connected component can be analytically computed for RaCInG from its kernel, as described in our accompanying mathematical paper^20^ and previous work of similar random graph models^21^

It can be derived by computing the largest solution to a given system of equations. If we denote by $x_{t}$ and $y_{t}$ the largest solutions to the following system of nonlinear equations:

$$1-x_{t}=\exp\left(-\sum_{k=1}^{\infty }\kappa \left(t,k\right)q_{k}x_{k}\right)\text{,}$$

$$1-y_{t}=\exp\left(-\sum_{k=1}^{\infty }\kappa \left(k,t\right)q_{k}y_{k}\right)\text{.}$$

Then, the size of the largest strongly connected component is given by

$$\sum_{t=1}^{\infty }x_{t}y_{t}q_{t}\text{.}$$

Moreover, the contribution of a fixed cell-type s to the largest strongly connected component is given by $x_{s}y_{s}q_{s}$. We solve the system of inequalities using the “root” function of the scipy.optimize package in Python. This package uses a modified version of Powell’s method^79^ for finding local minima of a function.

###### Degree centrality

There are two types of degree centrality in directed graphs: the in-degree and out-degree centrality. For each cell type the in-/out-degree centrality is defined as the expected in-/out-degree of said cell type (Figure S1C). This can be computed directly from the kernel, since it is known for RaCInG that the out-degree distribution^21^ of a given cell with type t follows a Poisson distribution with parameter

$$\sum_{k=1}^{\infty }\kappa \left(t,k\right)q_{k}\text{.}$$

Similarly, the in-degree distribution follows a Poisson distribution with parameter

$$\sum_{k=1}^{\infty }\kappa \left(k,t\right)q_{k}\text{.}$$

Therefore, since the expected value of a Poisson distribution is equal to its parameter, we can use these values as RaCInG’s in- and out-degree centrality fingerprints.

#### From directed to undirected features

Features from both the Monte Carlo and kernel methods are directed. Based on the TCGA case study it was decided to use undirected features instead of directed features. To compute these from the directed features, all directed counts with the same cell-types were accumulated. For example, in the case of direct communication the undirected feature Dir_A_B was obtained by computing κ(A,B)+κ(B,A).

#### C-matrix perturbation

To perturb the C-matrix of a given patient we first take the normal values $Q=\left(q_{1},q_{2},\ldots ,q_{8}\right)$ and derive from them two perturbed quantification vectors $Q^{\prime }=\left(q_{1}^{\prime },q_{2}^{\prime },\ldots ,q_{8}^{\prime }\right)$ and $Q^{\prime \prime }=\left(q_{1}^{\prime \prime },q_{2}^{\prime \prime },\ldots ,q_{8}^{\prime \prime }\right)$. These are defined through the system of equations

$$C^{\prime }=q_{1}+\ldots +q_{8}+0.9q_{9}\text{,}$$

$$q_{9}^{\prime }=\frac{0.9q_{9}}{C^{\prime }}\text{,}$$

$$\forall_{i\leq 8}:q_{i}^{\prime }=\frac{q_{i}}{C^{\prime }}\text{,}$$

and

$$C^{\prime \prime }=0.9\left(q_{1}+\ldots +q_{8}\right)+q_{9}\text{,}$$

$$q_{9}^{\prime \prime }=\frac{q_{9}}{C^{\prime \prime }}\text{,}$$

$$\forall_{i\leq 8}:q_{i}^{\prime \prime }=\frac{0.9q_{i}}{C^{\prime }}\text{.}$$

Here $Q^{\prime }$ depicts the situation where the tumor quantification is perturbed by maximally 10%, and $Q^{\prime \prime }$ the situation where the other cell-types are perturbed by maximally 10%.

For each dataset, after the perturbed cell-type matrices have been computed, we calculate the kernel of the patients and compare this with the original kernels where the C-matrix was not perturbed.

#### Normalization

To normalize, the pipelines for network generation and feature extraction (either kernel or Monte Carlo) were executed again, but this time in a setting where the distribution P was made uniform over its support. Hence, if one sets

$$c=\sum_{i=1}^{\infty }\sum_{j=1}^{\infty }1\left\{p_{ij}>0\right\}\text{,}$$

where 1{·} indicates the indicator function, then in the uniform runs a new probability distribution $\tilde{P}$ was used for the ligand-receptor interactions. In this distribution, the probability of an interaction between ligand i and receptor j occurring was given by

$${\tilde{p}}_{ij}=1\left\{p_{ij}>0\right\}/c\text{.}$$

All other parameters were kept the same as in the previous “standard” runs. Finally, if $f_{st}$ is the (average) feature value in the “standard” run and $f_{unif}$ is the same (average) feature value in the uniform run, then the normalized feature value was given by the fold change between these two runs, i.e.,

$$f_{norm}=\frac{f_{st}}{f_{unif}}\text{.}$$

One can identify $f_{norm}$ as the number of times a feature would appear more often in the networks generated with the actual input data compared to the networks generated with input data that disregarded the LR-quantification. A big advantage of this normalization procedure is its ability to place all feature values on the same footing regardless of the method they were computed by. No matter if $f_{norm}$ is computed through the Monte Carlo method or the kernel method, its interpretation and value range stay the same.

#### Average number of arcs

To compute the average number of arcs from a cell-type t to a cell-type s in order to generate the Circos plots (Figures 5A and 5B), we use the kernel value κ(t,s) and the corresponding C-matrix entries $q_{t}$ and $q_{s}$. Recall that κ(t,s)/N can be interpreted as the probability that an arc appears between two fixed cells of type t and s. Since there are (on average) $q_{t}N$ cells of type t and $q_{s}N$ cells of type s, this means that the average number of arcs between the two cell-types is given by $q_{t}q_{s}\kappa \left(t,s\right)\;N$. Hence, as (directed) interaction values we used $q_{t}q_{s}\kappa \left(t,s\right)$ in order have feature values independent of N. Note that these values are not normalized like the other feature values used in this paper, since such normalized values would not be interpretable in a Circos plot. Circos plots were produced using the online tool “circos”.^80^

#### Ligand-receptor probability of cell-types

To compute the conditional probability that a certain LR-pair caused the formation of an interaction, given the interaction is between two given cell-types we only used the LR-distribution P and the compatibility matrices L and R. The unconditional probability of LR-pair (i,j) appearing is given by $p_{ij}\text{.}$ To infer its contribution to a direct interaction between cell-type k and l, one first needs to know whether it connects these cell-types. The indicator of this event is given by L(k,i)R(l,j).

Now, since all interactions were sampled and paired independently, and uniformly at random, the conditional probability of LR-pair (i,j) connecting cell-types k and l was given by the LR-pair’s relative (probabilistic) weight when compared to the weights of all LR-pairs that can connect cell-types k and l. Thus, the conditional probability that LR-pair (i,j) formed a connection, given that it is a connection between cell-types k and l, is given by

$$p_{ij}^{\left(kl\right)}=\frac{p_{ij}L\left(k,i\right)R\left(l,j\right)}{\alpha_{i}\beta_{j\;}\kappa \left(k,l\right)}\text{.}$$

Here, $\alpha_{i}$, $\beta_{j}$ and κ are the constants and functions as given in the subsection on kernel computation. To compute the LR-probability for given cell-types over an entire group, these probabilities were taken for all patients in the group, and averaged. The largest of the resulting averages were depicted in the LR-interaction bar charts.

### Quantification and statistical analysis

In general, correlations were assessed with the Spearman rho correlation coefficient except for the robustness to prior knowledge on expressed ligands and receptors, where Pearson r correlation is used due to the presumed linear relationship between the original and perturbed kernels. To calculate correlations the scipy.stats.spearmanr and scipy.stats.pearsonr function in Python was used based on the 1.9.2 version of the Scipy package. In the results section it is indicated which correlation coefficient is used, and what correlations are deemed large. Correlations are deemed significant with a *p*-value smaller than 0.05 after Bonferroni correction.

Linear and logistic regression have been executed using version 1.3.2 of the scikit-learn package in Python. Linear regression was validated using 1000 rounds of cross-validation where 20 patients were randomly removed from the same and used as test values for the resulting linear model. R-squared and MSE values were reported. Logistic regression was trained on fingerprints from the SKCM and STAD TCGA dataset, using immune phenotype as prediction target. Specific fingerprints used are indicated in the main text. Model parameters were selected by measuring model accuracy on the training dataset through 30-fold cross validation. Model was tested on the NSCLC TCGA dataset and combined Gideauslanderpd1 with Kim dataset (with immune phenotype as target), and on the combined Gideauslanderpd1 with Kim dataset and Gideauslanderpd1on dataset (with immune response as target; IE mapped to response). Performance of the model was reported through a ROC-curve combined with AUC scores.

A two-sided Wilcoxon rank-sum test at significance level α=0.05 (after Bonferroni correction) was applied to test for differences between direct, wedge and triangle network fingerprints in two groups of patients in the case studies, since no prior distributional knowledge for each fingerprint was available. To apply the test the function scipy.stats.ranksums from the 1.9.2 version of the Scipy package in Python was used. If a statistical difference between two groups was observed for a feature, the fold-change between the average feature values of the groups was used to infer how much the empirical distributions of the two groups overlap.

A one-sided Wilcoxon rank-sum test at significance level α=0.05 was applied to test the difference between tumor contribution to the largest strongly connected component in fixed patient groups for a fixed value of λ. Since robustness of the group differences over a range of λ-values was assessed, no Bonferroni correction was applied between fixed values of λ.

A two-sided Wilcoxon signed-rank test at significance level α=0.05 was applied to test the difference between LR interactions in matched tumor vs. normal STAD TCGA samples.

Sample sizes are indicated in the captions of each figure, and quantitative results are reported in the main text, and dispersion measures as well as statistical methods used for each figure are indicated in the legend of each figure. No tests have been executed to determine whether data fits the assumptions of statistical methods, since only robust statistical measures were used that make no prior assumption on the distribution of the data.

## Supplemental information

Document S1. Figures S1–S6 and Tables S6 and S7

Table S1. Normalized kernel values for all datasets, related to STAR methods Sheet 1 - Normalized kernel values for all patients computed by RaCInG directly from its input. Sheet 2 - Kernel values for some patients computed by RaCInG after perturbing either the tumor cell quantification or all other cell quantifications by 10%. Sheet 3 - Kernel values computed by RaCInG from the output of CODEFACS. Sheet 4 - Values extracted from LIRICS that were compared with the kernel values from RaCInG.

Table S2. Unnormalized kernel values for all datasets, related to STAR methods Sheet 1 - Unnormalized kernel values for all patients in TCGA datasets. Sheet 2 - Unnormalized kernel values for all patients in Gide, Auslander, Kim and Wu cohorts. Sheet 3 - Unnormalized direct communication values for all patients in TCGA datasets. Sheet 4 - Unnormalized direct communication values for all patients in Gide, Auslander, Kim and Wu cohorts.

Table S3. All fingerprint values computed by RaCInG with metadata, related to STAR methods Sheet 1 - Wedge, triangle, direct communication and strongly connected component fingerprints for all patients in TCGA datasets with direction removed for each. Sheet 2 - Wedge, triangle, direct communication and strongly connected component fingerprints for all patients in Gide, Auslander, Kim and Wu cohorts with direction removed for each. Sheet 3 - Directed wedge, triangle, direct communication and strongly connected component fingerprints for all patients in Gide, Auslander, Kim and Wu cohorts.

Table S4. All available spatial colocalization scores for patients in the Wu dataset, related to STAR methods Each sheet represents a different patient from the dataset.

Table S5. Results from Monte-Carlo experiments, related to STAR methods Each sheet indicates a cancer type. The sheet is filled with (normalized) network fingerprints calculated with the Monte-Carlo method.

Document S2. Article plus supplemental information
