# Supplementary material for: Mathematically mapping the network of cells in the tumor microenvironment
Source: Cell Rep Methods. 2025 Feb 14;5(2):100985. doi: 10.1016/j.crmeth.2025.100985 (PMC11955271; doi:10.1016/j.crmeth.2025.100985)
Supplement: Document S1. Figures S1–S6 and Tables S6 and S7 [file mmc1.pdf]

Cell Reports Methods, Volume *5*

Supplemental information

Mathematically mapping the network of cells in the tumor microenvironment

Mike van Santvoort, Óscar Lapuente-Santana, Maria Zopoglou, Constantin Zackl, Francesca Finotello, Pim van der Hoorn, and Federica Eduati

Supplemental Information

Mathematically mapping the network of cells in the tumor microenvironment

Mike van Santvoort[1,2], Óscar Lapuente-Santana[2,3,4], Maria Zopoglou[5], Constantin Zackl[5], Francesca Finotello[5], Pim van der Hoorn[1,2,*], Federica Eduati[2,3,*,6]

1 Department of Mathematics and Computer Science, Eindhoven University of Technology, Eindhoven, PO Box 513, 5600MB, Eindhoven, The Netherlands.

2 Institute for Complex Molecular Systems, Eindhoven University of Technology, PO Box 513, 5600MB, Eindhoven, The Netherlands.

3 Department of Biomedical Engineering, Eindhoven University of Technology, PO Box 513, 5600MB, Eindhoven, The Netherlands.

4 Bioinformatics Unit, Spanish National Cancer Research Centre (CNIO), 28029 Madrid, Spain

5 Department of Molecular Biology, Digital Science Center (DiSC), University of Innsbruck, 6020 Innsbruck, Austria

6 Lead contact

* Co-corresponding authors: Federica Eduati f.eduati@tue.nl, Pim van der Hoorn w.l.f.v.d.hoorn@tue.nl

Table S6: Information associated with the datasets of patients treated with immunotherapy, related to STAR methods. FFPE: Formalin-fixed paraffin-embedded; FF: Fresh-frozen; CR: Complete Responder; PR: Partial Responder; PD: Progressive Disease; SD: Stable Disease; R: Responder; NR: Non-responder.

**Original study**	**Cancer type**	**Prior therapies**	**Biopsy**	**Samples used**	**R and NR**	**RNA-seq fastq files**
Auslander [1]	Melanoma (Metastasis)	Therapy naive	FF	PD-1:<br>- Pre: n=9 (R=1, NR=8)<br>- On: n=17 (R=0, NR=17)	As reported.	BioProject ID: PRJNA476140
Gide [2]	Melanoma (Metastasis)	BRAFi	FFPE	PD-1:<br>- Pre: n=41 (CR=4, PR=15, PD=16, SD=6)<br>- On: n=9 (CR=0, PR=4, PD=4, SD=1)	R=CR,PR<br>NR=SD,PD	BioProject ID: PRJEB23709
Kim [3]	Gastric cancer (Metastasis)	Prior failure of at least 1 line of chemotherapy (platinum)	FF	Pre: n=45 (CR=3, PR=9, PD=18, SD=15)	R=CR,PR<br>NR=SD,PD	BioProject ID: PRJEB25780

Table S7: Mapping between cell-type from deconvolution of spatial transcriptomics data and cell-type from deconvolution of bulk RNA-seq data, related to STAR methods.

Bulk cell-type	Spatial cell-type(s)
B	B.cells.Memory<br>B.cells.Naive
CAF	CAFs.MSC.iCAF.like<br>CAFs.myCAF.like
CD8+ T	T.cells.CD8
DC	DCs
Endo	Endothelial.ACKR1<br>Endothelial.CXCL12<br>Endothelial.Lymphatic.LYVE1<br>Endothelial.RGS5
M	Macrophage
NK	NK.cells; NKT.cells
Treg	T.cells.CD4
Tumor	Cancer.Basal.SC<br>Cancer.Her2.SC<br>Cancer.LumA.SC<br>Cancer.LumB.SC

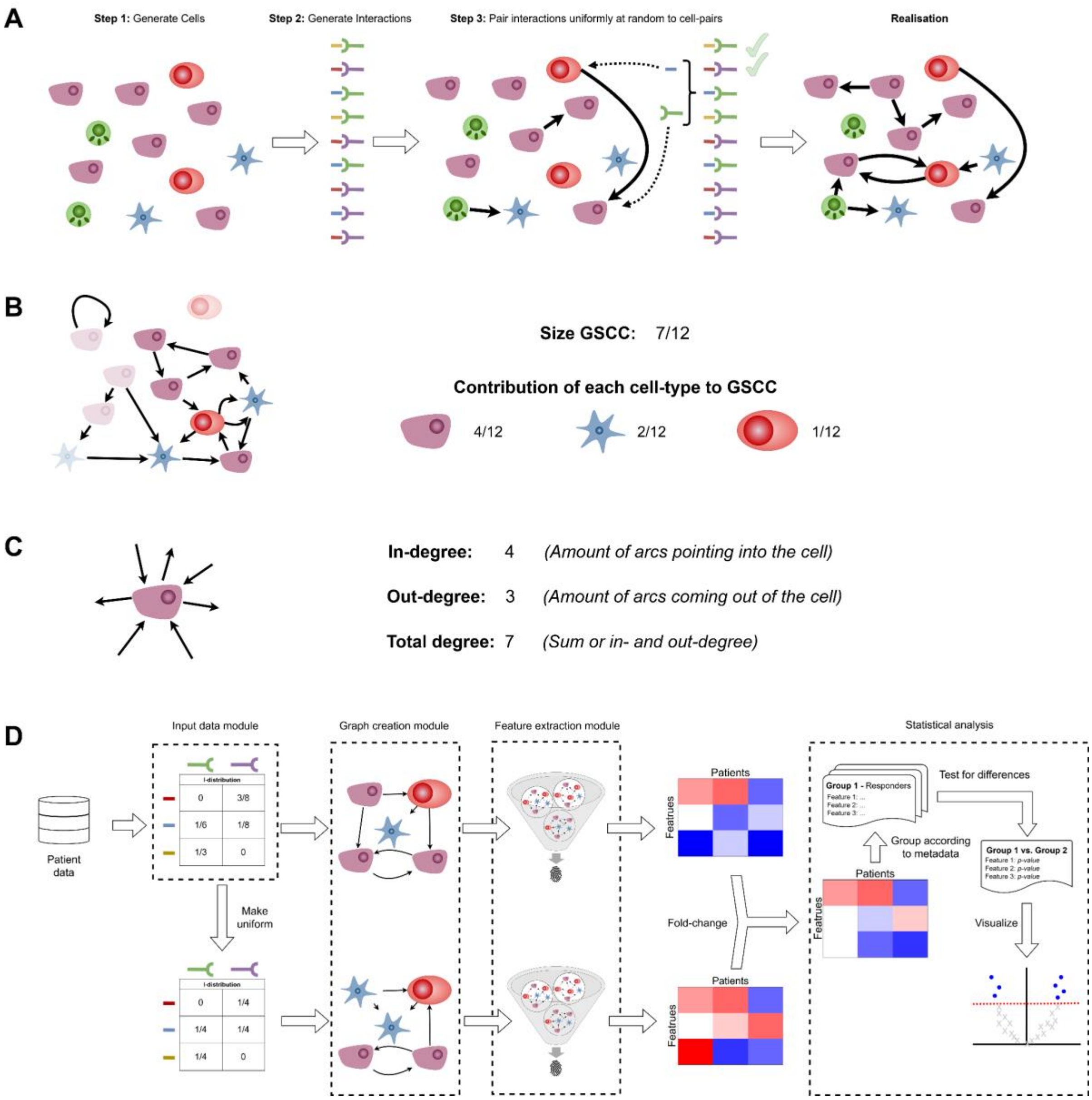

Figure S1: From input matrices to feature extraction, related to Figure 1. (A) A schematic overview of the monte-carlo method to generate an individual graph instance based on the four input matrices. (B) A visual example of the largest strongly connected component in a graph (opaque cells) together with its corresponding feature value (global and for each cell-type). (C) Visual example of the concept of in- and out-degree of a vertex in a directed graph (needed to compute in- and out-degree centrality). (D) Visual representation of the normalization procedure.

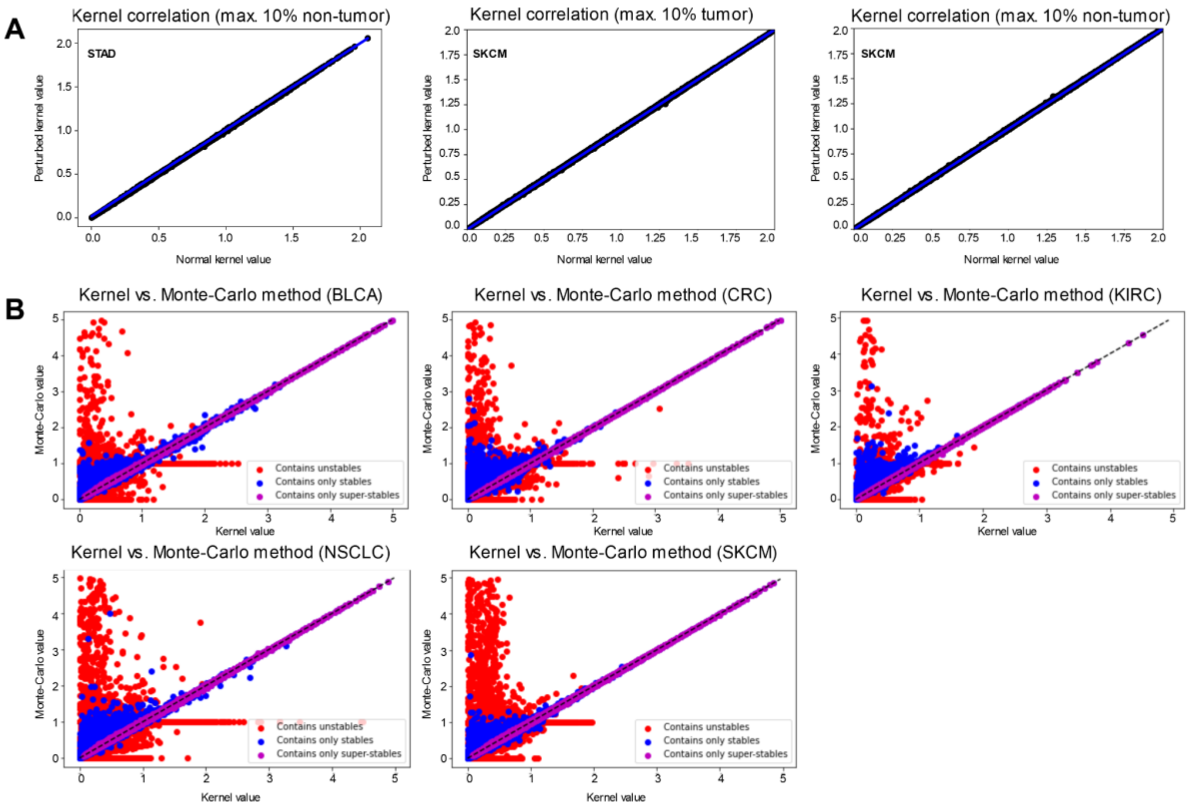

Figure S2: Additional robustness analyses, related to Figure 2. (A) Perturbing the C-distribution in RaCInG input. In the STAD TCGA dataset the non-tumor cells were perturbed directly, whilst only perturbing the tumor cell through re-normalization (max. 10% perturbation). Output MSE of 0.00016 and the maximum relative output error of 5.6%. In the SKCM dataset both tumor and non-tumor cells have been perturbed once directly (max. 10%), perturbing the other cells only through normalization. Output MSE smaller than 0.0001 and maximum relative output error of 8.4% when tumor cells were perturbed directly, and MSE smaller than 0.0001 and maximum relative output error or 6.5% when non-tumor cells were perturbed directly. (B) Agreement between monte-carlo and kernel method in RaCInG for other TCGA datasets.

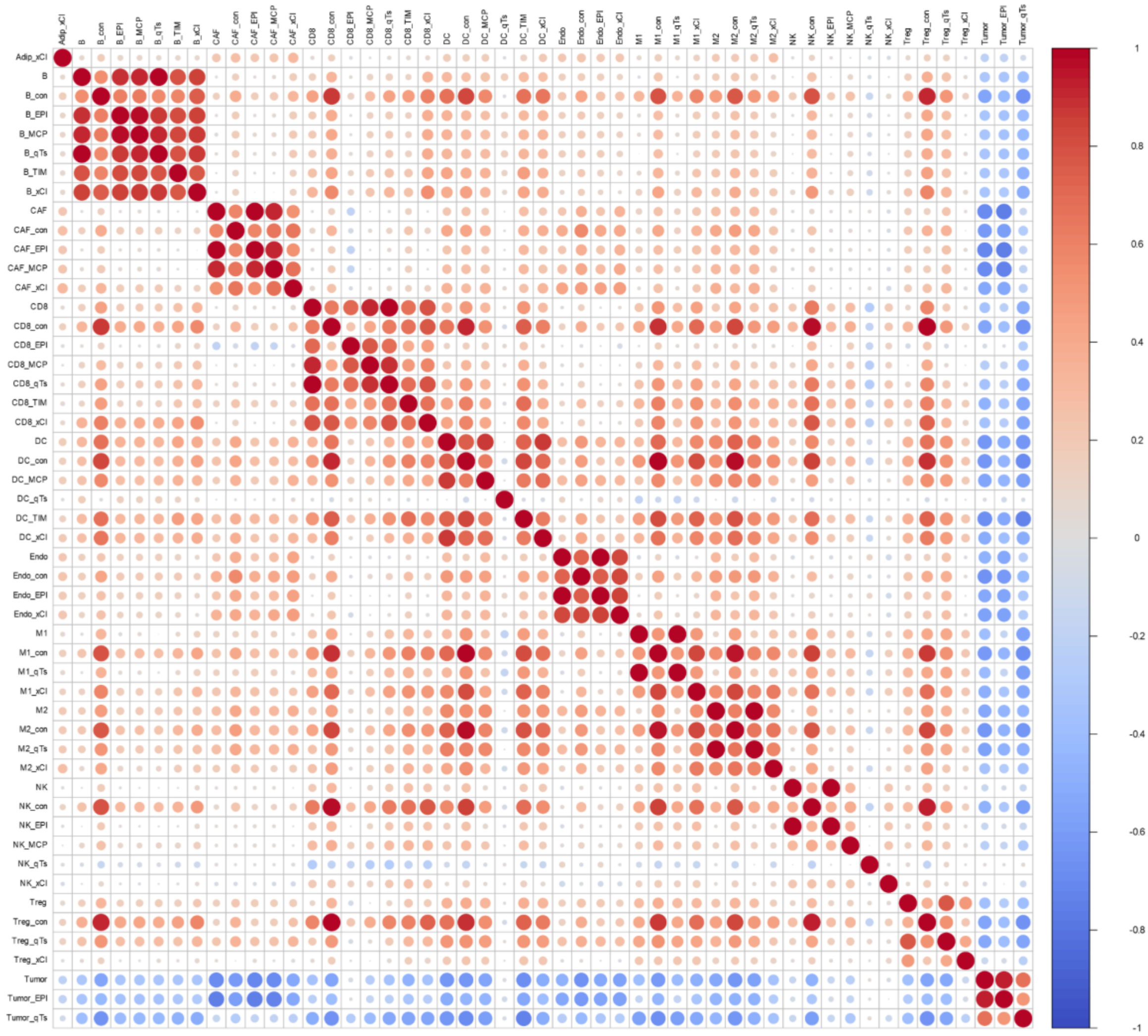

Figure S3: Agreement between deconvolution methods, related to Figure 3 and 4. Mean correlation (across TCGA cancer types) between cell-type quantification computed using multiple in silico deconvolution methods.

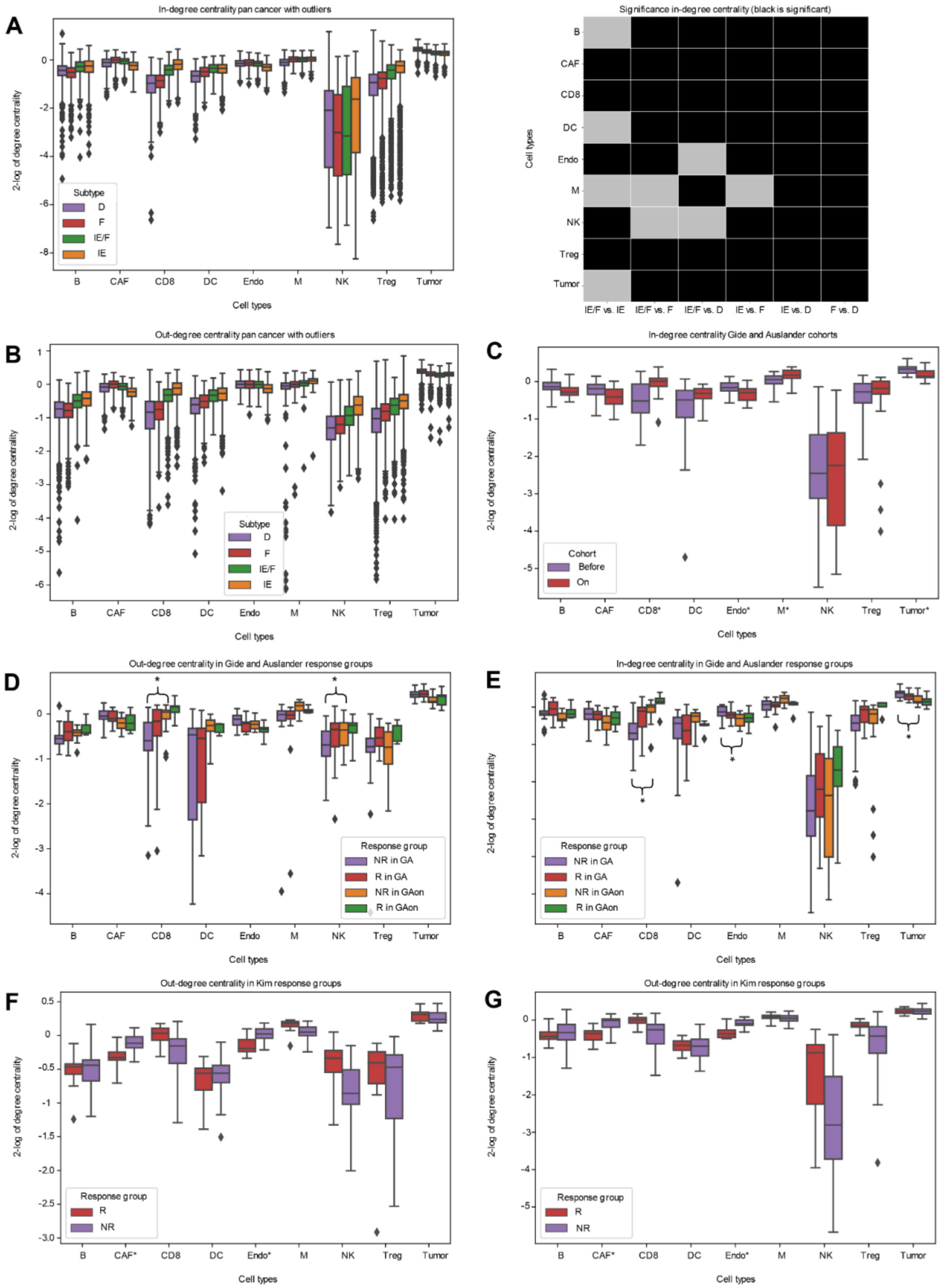

Figure S4: Additional centrality analyses, related to Figures 4 and 5. (A) Distribution of pan-cancer in-degree centralities, divided over MFP phenotypes (left) and the significance of the differences between these distributions (right; two-sided Mann-Whitney-U test at $\alpha = 0.05$). (B) Distribution of pan-cancer out-degree centralities showing the outliers. (C) Distribution of in-degree centrality of Gide and Auslander cohorts. Significant cell-types indicated with a star (two-sided Mann-Whitney-U test at $\alpha = 0.05$). (D, E) Out- and in-centrality distribution of Gide and Auslander cohort subdivided into response groups. Significant cell-types

indicated with a star (two-sided Mann-Whitney-U test at $\alpha = 0.05$). (F, G) Out- and in-degree centrality of kim cohort subdivided into response groups. Significant cell-types indicated with a star (two-sided Mann-Whitney-U test at $\alpha = 0.05$).

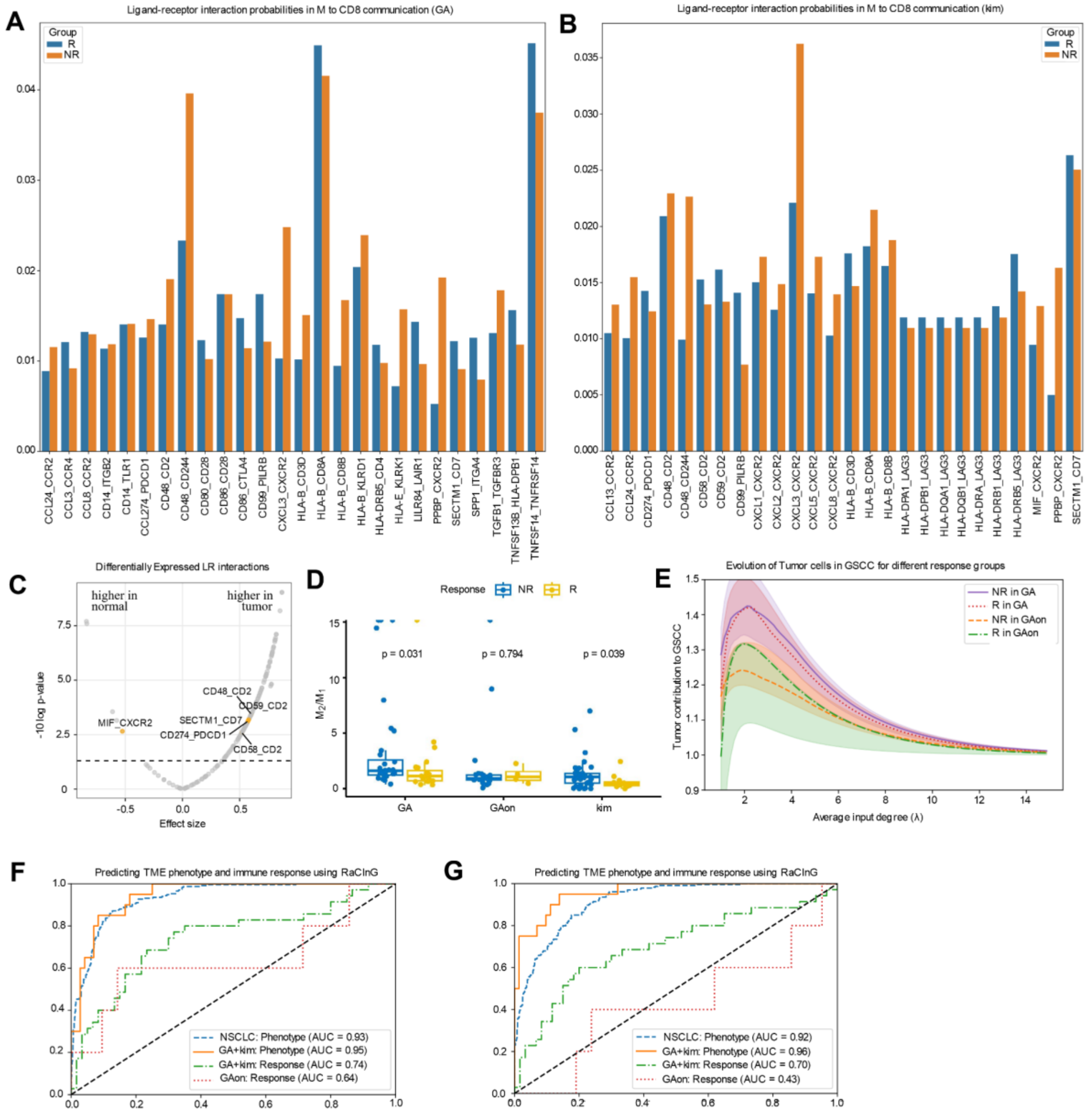

Figure S5: Additional figures for response to ICB analysis, related to Figure 5. (A) Protein communication scores between CD8+ T-cells to macrophages in the Gide-Auslander cohorts before treatment. Only the top 20 interactions for both groups are depicted. (B) Protein communication scores between CD8+ T-cells to macrophages in the Kim cohort. Only the top 20 interactions for both groups are depicted. (C) Differential expression of LR interactions in tumor vs. normal tissue using Wilcoxon signed-rank test at significance level $\alpha = 0.05$. The analysis is based on 67 matched samples from the STAD TCGA cohort. The top 20 LR interactions higher in NR in the Kim dataset have been highlighted in orange and marked with the corresponding LR name when appearing as among the significantly differentially expressed. (D) Cell-type quantification of macrophage phenotypes in patients of the Gide and Auslander cohorts before and on treatment. Each boxplot represents the macrophage quantification of a certain phenotype in a certain response group. The boxplots have been generated for the patients before and on immunotherapy. M1 macrophages have the anti-tumor phenotype while M2 macrophages the pro-tumor phenotype. (E) The evolution of the largest strongly connected component for different response groups before and on PD-L1 therapy. The shaded area indicates the data between Q1 and Q3 for all datasets. After applying the Mann-Whitney-U test for all pairwise connections and correcting for multiple hypothesis testing no significant comparisons were found at the significance level $\alpha = 0.05$. (F) ROC-curve for logistic regression model trained on on normalized kernel values (i.e., direct communication), wedge fingerprints and triangle fingerprints of SKCM and STAD datasets and tested on MFP

or immune response in NSCLC, Gide-Auslander and Kim datasets. (F) ROC-curve for logistic regression model trained on unnormalized kernel values.

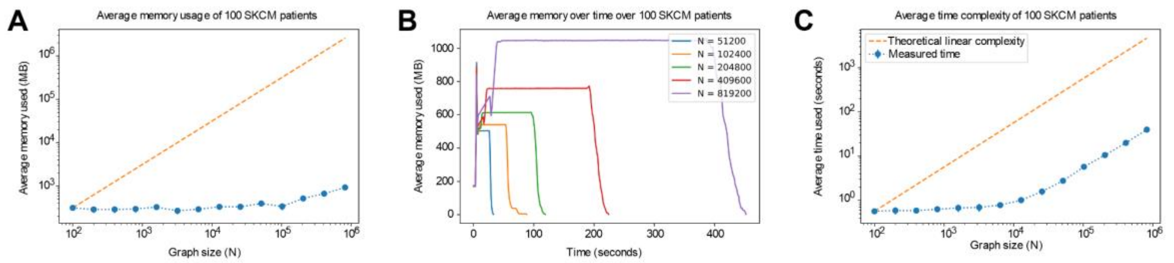

Figure S6: Time and memory measurements of the graph generation algorithm for increasing graph sizes, related to STAR methods. (A) Average memory usage over the entire run for 100 SKCM patients (with error bars encompassing two standard deviations). (B) Average memory usage at each time-point for one run of the graph-generation algorithm over 100 SKCM patients. (C) Average time complexity of one graph generation for 100 SKCM patients (with error bars encompassing two standard deviations).

1. Auslander, N. *et al.* Robust prediction of response to immune checkpoint blockade therapy in metastatic melanoma. *Nat. Med.* **24**, 1545–1549 (2018).
2. Gide, T. N. *et al.* Distinct Immune Cell Populations Define Response to Anti-PD-1 Monotherapy and Anti-PD-1/Anti-CTLA-4 Combined Therapy. *Cancer Cell* **35**, 238–255.e6 (2019).
3. Kim, S. T. *et al.* Comprehensive molecular characterization of clinical responses to PD-1 inhibition in metastatic gastric cancer. *Nat. Med.* **24**, 1449–1458 (2018).
4. Wu, S. Z. *et al.* A single-cell and spatially resolved atlas of human breast cancers. *Nat. Genet.* **53**, 1334–1347 (2021).
